# Measurements of angular distance and momentum ratio distributions in three-jet and ${\text {Z}}$ + two-jet final states in ${\text {p}}{\text {p}}$ collisions

**DOI:** 10.1140/epjc/s10052-021-09570-2

**Published:** 2021-09-27

**Authors:** A. M. Sirunyan, A. Tumasyan, W. Adam, T. Bergauer, M. Dragicevic, J. Erö, A. Escalante Del Valle, R. Frühwirth, M. Jeitler, N. Krammer, L. Lechner, D. Liko, T. Madlener, I. Mikulecc, F. M. Pitters, N. Rad, J. Schieck, R. Schöfbeck, M. Spanring, S. Templ, W. Waltenberger, C.-E. Wulz, M. Zarucki, V. Chekhovsky, A. Litomin, V. Makarenko, M. R. Darwish, E. A. De Wolf, D. Di Croce, X. Janssen, T. Kello, A. Lelek, M. Pieters, H. Rejeb Sfar, H. Van Haevermaet, P. Van Mechelen, S. Van Putte, N. Van Remortel, F. Blekman, E. S. Bols, S. S. Chhibra, J. D’Hondt, J. De Clercq, D. Lontkovskyi, S. Lowette, I. Marchesini, S. Moortgat, A. Morton, Q. Python, S. Tavernier, W. Van Doninck, P. Van Mulders, D. Beghin, B. Bilin, B. Clerbaux, G. De Lentdecker, B. Dorney, L. Favart, A. Grebenyuk, A. K. Kalsi, I. Makarenko, L. Moureaux, L. Pétré, A. Popov, N. Postiau, E. Starling, L. Thomas, C. Vander Velde, P. Vanlaer, D. Vannerom, L. Wezenbeek, T. Cornelis, D. Dobur, M. Gruchala, I. Khvastunov, M. Niedziela, C. Roskas, K. Skovpen, M. Tytgat, W. Verbeke, B. Vermassen, M. Vit, G. Bruno, F. Bury, C. Caputo, P. David, C. Delaere, M. Delcourt, I. S. Donertas, A. Giammanco, V. Lemaitre, K. Mondal, J. Prisciandaro, A. Taliercio, M. Teklishyn, P. Vischia, S. Wertz, S. Wuyckens, J. Zobec, G. A. Alves, C. Hensel, A. Moraes, W. L. Aldá Júnior, E. Belchior Batista Das Chagas, H. Brandao Malbouisson, W. Carvalho, J. Chinellato, E. Coelho, E. M. Da Costa, G. G. Da Silveira, D. De Jesus Damiao, S. Fonseca De Souza, J. Martins, D. Matos Figueiredo, M. Medina Jaime, C. Mora Herrera, L. Mundim, H. Nogima, P. Rebello Teles, L. J. Sanchez Rosas, A. Santoro, S. M. Silva Do Amaral, A. Sznajder, M. Thiel, F. Torres Da Silva De Araujo, A. Vilela Pereira, C. A. Bernardes, L. Calligaris, T. R. Fernandez Perez Tomei, E. M. Gregores, D. S. Lemos, P. G. Mercadante, S. F. Novaes, Sandra S. Padula, A. Aleksandrov, G. Antchev, I. Atanasov, R. Hadjiiska, P. Iaydjiev, M. Misheva, M. Rodozov, M. Shopova, G. Sultanov, M. Bonchev, A. Dimitrov, T. Ivanov, L. Litov, B. Pavlov, P. Petkov, A. Petrov, W. Fang, Q. Guo, H. Wang, L. Yuan, M. Ahmad, Z. Hu, Y. Wang, E. Chapon, G. M. Chen, H. S. Chen, M. Chen, T. Javaid, A. Kapoor, D. Leggat, H. Liao, Z. Liu, R. Sharma, A. Spiezia, J. Tao, J. Thomas-Wilsker, J. Wang, H. Zhang, S. Zhang, J. Zhao, A. Agapitos, Y. Ban, C. Chen, Q. Huang, A. Levin, Q. Li, M. Lu, X. Lyu, Y. Mao, S. J. Qian, D. Wang, Q. Wang, J. Xiao, Z. You, X. Gao, M. Xiao, C. Avila, A. Cabrera, C. Florez, J. Fraga, A. Sarkar, M. A. Segura Delgado, J. Jaramillo, J. Mejia Guisao, F. Ramirez, J. D. Ruiz Alvarez, C. A. Salazar González, N. Vanegas Arbelaez, D. Giljanovic, N. Godinovic, D. Lelas, I. Puljak, T. Sculac, Z. Antunovic, M. Kovac, V. Brigljevic, D. Ferencek, D. Majumder, M. Roguljic, A. Starodumov, T. Susa, M. W. Ather, A. Attikis, E. Erodotou, A. Ioannou, G. Kole, M. Kolosova, S. Konstantinou, G. Mavromanolakis, J. Mousa, C. Nicolaou, F. Ptochos, P. A. Razis, H. Rykaczewski, H. Saka, D. Tsiakkouri, M. Finger, M. Finger, A. Kveton, J. Tomsa, E. Ayala, E. Carrera Jarrin, H. Abdalla, A. A. Abdelalim, S. Elgammal, M. A. Mahmoud, Y. Mohammed, S. Bhowmik, A. Carvalho Antunes De Oliveira, R. K. Dewanjee, K. Ehataht, M. Kadastik, M. Raidal, C. Veelken, P. Eerola, L. Forthomme, H. Kirschenmann, K. Osterberg, M. Voutilainen, E. Brücken, F. Garcia, J. Havukainen, V. Karimäki, M. S. Kim, R. Kinnunen, T. Lampén, K. Lassila-Perini, S. Lehti, T. Lindén, H. Siikonen, E. Tuominen, J. Tuominiemi, P. Luukka, T. Tuuva, C. Amendola, M. Besancon, F. Couderc, M. Dejardin, D. Denegri, J. L. Faure, F. Ferri, S. Ganjour, A. Givernaud, P. Gras, G. Hamel de Monchenault, P. Jarry, B. Lenzi, E. Locci, J. Malcles, J. Rander, A. Rosowsky, M. Ö. Sahin, A. Savoy-Navarro, M. Titov, G. B. Yu, S. Ahuja, F. Beaudette, M. Bonanomi, A. Buchot Perraguin, P. Busson, C. Charlot, O. Davignon, B. Diab, G. Falmagne, R. Granier de Cassagnac, A. Hakimi, I. Kucher, A. Lobanov, C. Martin Perez, M. Nguyen, C. Ochando, P. Paganini, J. Rembser, R. Salerno, J. B. Sauvan, Y. Sirois, A. Zabi, A. Zghiche, J.-L. Agram, J. Andrea, D. Bloch, G. Bourgatte, J.-M. Brom, E. C. Chabert, C. Collard, J.-C. Fontaine, D. Gelé, U. Goerlach, C. Grimault, A.-C. Le Bihan, P. Van Hove, E. Asilar, S. Beauceron, C. Bernet, G. Boudoul, C. Camen, A. Carle, N. Chanon, D. Contardo, P. Depasse, H. El Mamouni, J. Fay, S. Gascon, M. Gouzevitch, B. Ille, Sa. Jain, I. B. Laktineh, H. Lattaud, A. Lesauvage, M. Lethuillier, L. Mirabito, L. Torterotot, G. Touquet, M. Vander Donckt, S. Viret, G. Adamov, Z. Tsamalaidze, L. Feld, K. Klein, M. Lipinski, D. Meuser, A. Pauls, M. Preuten, M. P. Rauch, J. Schulz, M. Teroerde, D. Eliseev, M. Erdmann, P. Fackeldey, B. Fischer, S. Ghosh, T. Hebbeker, K. Hoepfner, H. Keller, L. Mastrolorenzo, M. Merschmeyer, A. Meyer, G. Mocellin, S. Mondal, S. Mukherjee, D. Noll, A. Novak, T. Pook, A. Pozdnyakov, Y. Rath, H. Reithler, J. Roemer, A. Schmidt, S. C. Schuler, A. Sharma, S. Wiedenbeck, S. Zaleski, C. Dziwok, G. Flügge, W. Haj Ahmad, O. Hlushchenko, T. Kress, A. Nowack, C. Pistone, O. Pooth, D. Roy, H. Sert, A. Stahl, T. Ziemons, H. Aarup Petersen, M. Aldaya Martin, P. Asmuss, I. Babounikau, S. Baxter, O. Behnke, A. Bermúdez Martínez, A. A. Bin Anuar, K. Borras, V. Botta, D. Brunner, A. Campbell, A. Cardini, P. Connor, S. Consuegra Rodríguez, V. Danilov, A. De Wit, M. M. Defranchis, L. Didukh, D. Domínguez Damiani, G. Eckerlin, D. Eckstein, T. Eichhorn, L. I. Estevez Banos, E. Gallo, A. Geiser, A. Giraldi, A. Grohsjean, M. Guthoff, A. Harb, A. Jafari, N. Z. Jomhari, H. Jung, A. Kasem, M. Kasemann, H. Kaveh, C. Kleinwort, J. Knolle, D. Krücker, W. Lange, T. Lenz, J. Lidrych, K. Lipka, W. Lohmann, R. Mankel, I.-A. Melzer-Pellmann, J. Metwally, A. B. Meyer, M. Meyer, M. Missiroli, J. Mnich, A. Mussgiller, V. Myronenko, Y. Otarid, D. Pérez Adán, S. K. Pflitsch, D. Pitzl, A. Raspereza, A. Saggio, A. Saibel, M. Savitskyi, V. Scheurer, C. Schwanenberger, A. Singh, R. E. Sosa Ricardo, N. Tonon, O. Turkot, A. Vagnerini, M. Van De Klundert, R. Walsh, D. Walter, Y. Wen, K. Wichmann, C. Wissing, S. Wuchterl, O. Zenaiev, R. Zlebcik, R. Aggleton, S. Bein, L. Benato, A. Benecke, K. De Leo, T. Dreyer, A. Ebrahimi, M. Eich, F. Feindt, A. Fröhlich, C. Garbers, E. Garutti, P. Gunnellini, J. Haller, A. Hinzmann, A. Karavdina, G. Kasieczka, R. Klanner, R. Kogler, V. Kutzner, J. Lange, T. Lange, A. Malara, C. E. N. Niemeyer, A. Nigamova, K. J. Pena Rodriguez, O. Rieger, P. Schleper, S. Schumann, J. Schwandt, D. Schwarz, J. Sonneveld, H. Stadie, G. Steinbrück, B. Vormwald, I. Zoi, S. Baur, J. Bechtel, T. Berger, E. Butz, R. Caspart, T. Chwalek, W. De Boer, A. Dierlamm, A. Droll, K. El Morabit, N. Faltermann, K. Flöh, M. Giffels, A. Gottmann, F. Hartmann, C. Heidecker, U. Husemann, M. A. Iqbal, I. Katkov, P. Keicher, R. Koppenhöfer, S. Maier, M. Metzler, S. Mitra, D. Müller, Th. Müller, M. Musich, G. Quast, K. Rabbertz, J. Rauser, D. Savoiu, D. Schäfer, M. Schnepf, M. Schröder, D. Seith, I. Shvetsov, H. J. Simonis, R. Ulrich, M. Wassmer, M. Weber, R. Wolf, S. Wozniewski, G. Anagnostou, P. Asenov, G. Daskalakis, T. Geralis, A. Kyriakis, D. Loukas, G. Paspalaki, A. Stakia, M. Diamantopoulou, D. Karasavvas, G. Karathanasis, P. Kontaxakis, C. K. Koraka, A. Manousakis-Katsikakis, A. Panagiotou, I. Papavergou, N. Saoulidou, K. Theofilatos, K. Vellidis, E. Vourliotis, G. Bakas, K. Kousouris, I. Papakrivopoulos, G. Tsipolitis, A. Zacharopoulou, I. Evangelou, C. Foudas, P. Gianneios, P. Katsoulis, P. Kokkas, K. Manitara, N. Manthos, I. Papadopoulos, J. Strologas, M. Bartók, R. Chudasama, M. Csanad, M. M. A. Gadallah, S. Lökös, P. Major, K. Mandal, A. Mehta, G. Pasztor, O. Surányi, G. I. Veres, G. Bencze, C. Hajdu, D. Horvath, F. Sikler, V. Veszpremi, G. Vesztergombi, S. Czellar, J. Karancsi, J. Molnar, Z. Szillasi, D. Teyssier, P. Raics, Z. L. Trocsanyi, B. Ujvari, T. Csorgo, F. Nemes, T. Novak, S. Choudhury, J. R. Komaragiri, D. Kumar, L. Panwar, P. C. Tiwari, S. Bahinipati, D. Dash, C. Kar, P. Mal, T. Mishra, V. K. Muraleedharan Nair Bindhu, A. Nayak, D. K. Sahoo, N. Sur, S. K. Swain, S. Bansal, S. B. Beri, V. Bhatnagar, S. Chauhan, N. Dhingra, R. Gupta, A. Kaur, S. Kaur, P. Kumari, M. Meena, K. Sandeep, S. Sharma, J. B. Singh, A. K. Virdi, A. Ahmed, A. Bhardwaj, B. C. Choudhary, R. B. Garg, M. Gola, S. Keshri, A. Kumar, M. Naimuddin, P. Priyanka, K. Ranjan, A. Shah, M. Bharti, R. Bhattacharya, S. Bhattacharya, D. Bhowmik, S. Dutta, S. Ghosh, B. Gomber, M. Maity, S. Nandan, P. Palit, P. K. Rout, G. Saha, B. Sahu, S. Sarkar, M. Sharan, B. Singh, S. Thakur, P. K. Behera, S. C. Behera, P. Kalbhor, A. Muhammad, R. Pradhan, P. R. Pujahari, A. Sharma, A. K. Sikdar, D. Dutta, V. Kumar, K. Naskar, P. K. Netrakanti, L. M. Pant, P. Shukla, T. Aziz, M. A. Bhat, S. Dugad, R. Kumar Verma, G. B. Mohanty, U. Sarkar, S. Banerjee, S. Bhattacharya, S. Chatterjee, M. Guchait, S. Karmakar, S. Kumar, G. Majumder, K. Mazumdar, S. Mukherjee, D. Roy, S. Dube, B. Kansal, S. Pandey, A. Rane, A. Rastogi, S. Sharma, H. Bakhshiansohi, M. Zeinali, S. Chenarani, S. M. Etesami, M. Khakzad, M. Mohammadi Najafabadi, M. Felcini, M. Grunewald, M. Abbrescia, R. Aly, C. Aruta, A. Colaleo, D. Creanza, N. De Filippis, M. De Palma, A. Di Florio, A. Di Pilato, W. Elmetenawee, L. Fiore, A. Gelmi, M. Gul, G. Iaselli, M. Ince, S. Lezki, G. Maggi, M. Maggi, I. Margjeka, V. Mastrapasqua, J. A. Merlin, S. My, S. Nuzzo, A. Pompili, G. Pugliese, A. Ranieri, G. Selvaggi, L. Silvestris, F. M. Simone, R. Venditti, P. Verwilligen, G. Abbiendi, C. Battilana, D. Bonacorsi, L. Borgonovi, S. Braibant-Giacomelli, R. Campanini, P. Capiluppi, A. Castro, F. R. Cavallo, C. Ciocca, M. Cuffiani, G. M. Dallavalle, T. Diotalevi, F. Fabbri, A. Fanfani, E. Fontanesi, P. Giacomelli, C. Grandi, L. Guiducci, F. Iemmi, S. Lo Meo, S. Marcellini, G. Masetti, F. L. Navarria, A. Perrotta, F. Primavera, A. M. Rossi, T. Rovelli, G. P. Siroli, N. Tosi, S. Albergo, S. Costa, A. Di Mattia, R. Potenza, A. Tricomi, C. Tuve, G. Barbagli, A. Cassese, R. Ceccarelli, V. Ciulli, C. Civinini, R. D’Alessandro, F. Fiori, E. Focardi, G. Latino, P. Lenzi, M. Lizzo, M. Meschini, S. Paoletti, R. Seidita, G. Sguazzoni, L. Viliani, L. Benussi, S. Bianco, D. Piccolo, M. Bozzo, F. Ferro, R. Mulargia, E. Robutti, S. Tosi, A. Benaglia, A. Beschi, F. Brivio, F. Cetorelli, V. Ciriolo, F. De Guio, M. E. Dinardo, P. Dini, S. Gennai, A. Ghezzi, P. Govoni, L. Guzzi, M. Malberti, S. Malvezzi, D. Menasce, F. Monti, L. Moroni, M. Paganoni, D. Pedrini, S. Ragazzi, T. Tabarelli de Fatis, D. Valsecchi, D. Zuolo, S. Buontempo, N. Cavallo, A. De Iorio, F. Fabozzi, F. Fienga, A. O. M. Iorio, L. Lista, S. Meola, P. Paolucci, B. Rossi, C. Sciacca, E. Voevodina, P. Azzi, N. Bacchetta, D. Bisello, A. Boletti, P. Bortignon, A. Bragagnolo, R. Carlin, P. Checchia, P. De Castro Manzano, T. Dorigo, F. Gasparini, U. Gasparini, S. Y. Hoh, L. Layer, M. Margoni, A. T. Meneguzzo, M. Presilla, P. Ronchese, R. Rossin, F. Simonetto, G. Strong, A. Tiko, M. Tosi, H. YARAR, M. Zanetti, P. Zotto, A. Zucchetta, G. Zumerle, C. Aime‘, A. Braghieri, S. Calzaferri, D. Fiorina, P. Montagna, S. P. Ratti, V. Re, M. Ressegotti, C. Riccardi, P. Salvini, I. Vai, P. Vitulo, M. Biasini, G. M. Bilei, D. Ciangottini, L. Fanò, P. Lariccia, G. Mantovani, V. Mariani, M. Menichelli, F. Moscatelli, A. Piccinelli, A. Rossi, A. Santocchia, D. Spiga, T. Tedeschi, K. Androsov, P. Azzurri, G. Bagliesi, V. Bertacchi, L. Bianchini, T. Boccali, R. Castaldi, M. A. Ciocci, R. Dell’Orso, M. R. Di Domenico, S. Donato, L. Giannini, A. Giassi, M. T. Grippo, F. Ligabue, E. Manca, G. Mandorli, A. Messineo, F. Palla, G. Ramirez-Sanchez, A. Rizzi, G. Rolandi, S. Roy Chowdhury, A. Scribano, N. Shafiei, P. Spagnolo, R. Tenchini, G. Tonelli, N. Turini, A. Venturi, P. G. Verdini, F. Cavallari, M. Cipriani, D. Del Re, E. Di Marco, M. Diemoz, E. Longo, P. Meridiani, G. Organtini, F. Pandolfi, R. Paramatti, C. Quaranta, S. Rahatlou, C. Rovelli, F. Santanastasio, L. Soffi, R. Tramontano, N. Amapane, R. Arcidiacono, S. Argiro, M. Arneodo, N. Bartosik, R. Bellan, A. Bellora, C. Biino, A. Cappati, N. Cartiglia, S. Cometti, M. Costa, R. Covarelli, N. Demaria, B. Kiani, F. Legger, C. Mariotti, S. Maselli, E. Migliore, V. Monaco, E. Monteil, M. Monteno, M. M. Obertino, G. Ortona, L. Pacher, N. Pastrone, M. Pelliccioni, G. L. Pinna Angioni, M. Ruspa, R. Salvatico, F. Siviero, V. Sola, A. Solano, D. Soldi, A. Staiano, D. Trocino, S. Belforte, V. Candelise, M. Casarsa, F. Cossutti, A. Da Rold, G. Della Ricca, F. Vazzoler, S. Dogra, C. Huh, B. Kim, D. H. Kim, G. N. Kim, J. Lee, S. W. Lee, C. S. Moon, Y. D. Oh, S. I. Pak, B. C. Radburn-Smith, S. Sekmen, Y. C. Yang, H. Kim, D. H. Moon, B. Francois, T. J. Kim, J. Park, S. Cho, S. Choi, Y. Go, S. Ha, B. Hong, K. Lee, K. S. Lee, J. Lim, J. Park, S. K. Park, J. Yoo, J. Goh, A. Gurtu, H. S. Kim, Y. Kim, J. Almond, J. H. Bhyun, J. Choi, S. Jeon, J. Kim, J. S. Kim, S. Ko, H. Kwon, H. Lee, K. Lee, S. Lee, K. Nam, B. H. Oh, M. Oh, S. B. Oh, H. Seo, U. K. Yang, I. Yoon, D. Jeon, J. H. Kim, B. Ko, J. S. H. Lee, I. C. Park, Y. Roh, D. Song, I. J. Watson, H. D. Yoo, Y. Choi, C. Hwang, Y. Jeong, H. Lee, Y. Lee, I. Yu, V. Veckalns, A. Juodagalvis, A. Rinkevicius, G. Tamulaitis, W. A. T. Wan Abdullah, M. N. Yusli, Z. Zolkapli, J. F. Benitez, A. Castaneda Hernandez, J. A. Murillo Quijada, L. Valencia Palomo, G. Ayala, H. Castilla-Valdez, E. De La Cruz-Burelo, I. Heredia-De La Cruz, R. Lopez-Fernandez, C. A. Mondragon Herrera, D. A. Perez Navarro, A. Sanchez-Hernandez, S. Carrillo Moreno, C. Oropeza Barrera, M. Ramirez-Garcia, F. Vazquez Valencia, J. Eysermans, I. Pedraza, H. A. Salazar Ibarguen, C. Uribe Estrada, A. Morelos Pineda, J. Mijuskovic, N. Raicevic, D. Krofcheck, S. Bheesette, P. H. Butler, A. Ahmad, M. I. Asghar, A. Awais, M. I. M. Awan, H. R. Hoorani, W. A. Khan, M. A. Shah, M. Shoaib, M. Waqas, V. Avati, L. Grzanka, M. Malawski, H. Bialkowska, M. Bluj, B. Boimska, T. Frueboes, M. Górski, M. Kazana, M. Szleper, P. Traczyk, P. Zalewski, K. Bunkowski, A. Byszuk, K. Doroba, A. Kalinowski, M. Konecki, J. Krolikowski, M. Olszewski, M. Walczak, M. Araujo, P. Bargassa, D. Bastos, P. Faccioli, M. Gallinaro, J. Hollar, N. Leonardo, T. Niknejad, J. Seixas, K. Shchelina, O. Toldaiev, J. Varela, V. Alexakhin, P. Bunin, M. Gavrilenko, A. Golunov, A. Golunov, I. Golutvin, I. Gorbunov, A. Kamenev, V. Karjavine, V. Korenkov, A. Lanev, A. Malakhov, V. Matveev, V. Palichik, V. Perelygin, M. Savina, S. Shmatov, S. Shulha, V. Smirnov, O. Teryaev, N. Voytishin, A. Zarubin, G. Gavrilov, V. Golovtcov, Y. Ivanov, V. Kim, E. Kuznetsova, V. Murzin, V. Oreshkin, I. Smirnov, D. Sosnov, V. Sulimov, L. Uvarov, S. Volkov, A. Vorobyev, Yu. Andreev, A. Dermenev, S. Gninenko, N. Golubev, A. Karneyeu, M. Kirsanov, N. Krasnikov, A. Pashenkov, G. Pivovarov, D. Tlisov, A. Toropin, V. Epshteyn, V. Gavrilov, N. Lychkovskaya, A. Nikitenko, V. Popov, G. Safronov, A. Spiridonov, A. Stepennov, M. Toms, E. Vlasov, A. Zhokin, T. Aushev, O. Bychkova, M. Chadeeva, D. Philippov, E. Popova, V. Rusinov, V. Andreev, M. Azarkin, I. Dremin, M. Kirakosyan, A. Terkulov, A. Belyaev, E. Boos, M. Dubinin, L. Dudko, A. Ershov, A. Gribushin, V. Klyukhin, O. Kodolova, I. Lokhtin, S. Obraztsov, S. Petrushanko, V. Savrin, A. Snigirev, V. Blinov, T. Dimova, L. Kardapoltsev, I. Ovtin, Y. Skovpen, I. Azhgirey, I. Bayshev, V. Kachanov, A. Kalinin, D. Konstantinov, V. Petrov, R. Ryutin, A. Sobol, S. Troshin, N. Tyurin, A. Uzunian, A. Volkov, A. Babaev, A. Iuzhakov, V. Okhotnikov, L. Sukhikh, V. Borchsh, V. Ivanchenko, E. Tcherniaev, P. Adzic, P. Cirkovic, M. Dordevic, P. Milenovic, J. Milosevic, M. Aguilar-Benitez, J. Alcaraz Maestre, A. Álvarez Fernández, I. Bachiller, M. Barrio Luna, Cristina F. Bedoya, J. A. Brochero Cifuentes, C. A. Carrillo Montoya, M. Cepeda, M. Cerrada, N. Colino, B. De La Cruz, A. Delgado Peris, J. P. Fernández Ramos, J. Flix, M. C. Fouz, A. García Alonso, O. Gonzalez Lopez, S. Goy Lopez, J. M. Hernandez, M. I. Josa, J. León Holgado, D. Moran, Á. Navarro Tobar, A. Pérez-Calero Yzquierdo, J. Puerta Pelayo, I. Redondo, L. Romero, S. Sánchez Navas, M. S. Soares, A. Triossi, L. Urda Gómez, C. Willmott, C. Albajar, J. F. de Trocóniz, R. Reyes-Almanza, B. Alvarez Gonzalez, J. Cuevas, C. Erice, J. Fernandez Menendez, S. Folgueras, I. Gonzalez Caballero, E. Palencia Cortezon, C. Ramón Álvarez, J. Ripoll Sau, V. Rodríguez Bouza, S. Sanchez Cruz, A. Trapote, I. J. Cabrillo, A. Calderon, B. Chazin Quero, J. Duarte Campderros, M. Fernandez, P. J. Fernández Manteca, G. Gomez, C. Martinez Rivero, P. Martinez Ruiz del Arbol, F. Matorras, J. Piedra Gomez, C. Prieels, F. Ricci-Tam, T. Rodrigo, A. Ruiz-Jimeno, L. Scodellaro, I. Vila, J. M. Vizan Garcia, M. K. Jayananda, B. Kailasapathy, D. U. J. Sonnadara, D. D. C. Wickramarathna, W. G. D. Dharmaratna, K. Liyanage, N. Perera, N. Wickramage, T. K. Aarrestad, D. Abbaneo, B. Akgun, E. Auffray, G. Auzinger, J. Baechler, P. Baillon, A. H. Ball, D. Barney, J. Bendavid, N. Beni, M. Bianco, A. Bocci, E. Bossini, E. Brondolin, T. Camporesi, G. Cerminara, L. Cristella, D. d’Enterria, A. Dabrowski, N. Daci, V. Daponte, A. David, A. De Roeck, M. Deile, R. Di Maria, M. Dobson, M. Dünser, N. Dupont, A. Elliott-Peisert, N. Emriskova, F. Fallavollita, D. Fasanella, S. Fiorendi, A. Florent, G. Franzoni, J. Fulcher, W. Funk, S. Giani, D. Gigi, K. Gill, F. Glege, L. Gouskos, M. Guilbaud, D. Gulhan, M. Haranko, J. Hegeman, Y. Iiyama, V. Innocente, T. James, P. Janot, J. Kaspar, J. Kieseler, M. Komm, N. Kratochwil, C. Lange, S. Laurila, P. Lecoq, K. Long, C. Lourenço, L. Malgeri, S. Mallios, M. Mannelli, A. Massironi, F. Meijers, S. Mersi, E. Meschi, F. Moortgat, M. Mulders, J. Niedziela, S. Orfanelli, L. Orsini, F. Pantaleo, L. Pape, E. Perez, M. Peruzzi, A. Petrilli, G. Petrucciani, A. Pfeiffer, M. Pierini, T. Quast, D. Rabady, A. Racz, M. Rieger, M. Rovere, H. Sakulin, J. Salfeld-Nebgen, S. Scarfi, C. Schäfer, C. Schwick, M. Selvaggi, A. Sharma, P. Silva, W. Snoeys, P. Sphicas, S. Summers, V. R. Tavolaro, D. Treille, A. Tsirou, G. P. Van Onsem, A. Vartak, M. Verzetti, K. A. Wozniak, W. D. Zeuner, L. Caminada, W. Erdmann, R. Horisberger, Q. Ingram, H. C. Kaestli, D. Kotlinski, U. Langenegger, T. Rohe, M. Backhaus, P. Berger, A. Calandri, N. Chernyavskaya, A. De Cosa, G. Dissertori, M. Dittmar, M. Donegà, C. Dorfer, T. Gadek, T. A. Gómez Espinosa, C. Grab, D. Hits, W. Lustermann, A.-M. Lyon, R. A. Manzoni, M. T. Meinhard, F. Micheli, F. Nessi-Tedaldi, F. Pauss, V. Perovic, G. Perrin, L. Perrozzi, S. Pigazzini, M. G. Ratti, M. Reichmann, C. Reissel, T. Reitenspiess, B. Ristic, D. Ruini, D. A. Sanz Becerra, M. Schönenberger, V. Stampf, M. L. Vesterbacka Olsson, R. Wallny, D. H. Zhu, C. Amsler, C. Botta, D. Brzhechko, M. F. Canelli, R. Del Burgo, J. K. Heikkilä, M. Huwiler, A. Jofrehei, B. Kilminster, S. Leontsinis, A. Macchiolo, P. Meiring, V. M. Mikuni, U. Molinatti, I. Neutelings, G. Rauco, A. Reimers, P. Robmann, K. Schweiger, Y. Takahashi, C. Adloff, C. M. Kuo, W. Lin, A. Roy, T. Sarkar, S. S. Yu, L. Ceard, P. Chang, Y. Chao, K. F. Chen, P. H. Chen, W.-S. Hou, Y. y. Li, R.-S. Lu, E. Paganis, A. Psallidas, A. Steen, E. Yazgan, B. Asavapibhop, C. Asawatangtrakuldee, N. Srimanobhas, F. Boran, S. Damarseckin, Z. S. Demiroglu, F. Dolek, C. Dozen, I. Dumanoglu, E. Eskut, G. Gokbulut, Y. Guler, E. Gurpinar Guler, I. Hos, C. Isik, E. E. Kangal, O. Kara, A. Kayis Topaksu, U. Kiminsu, G. Onengut, K. Ozdemir, A. Polatoz, A. E. Simsek, B. Tali, U. G. Tok, S. Turkcapar, I. S. Zorbakir, C. Zorbilmez, B. Isildak, G. Karapinar, K. Ocalan, M. Yalvac, I. O. Atakisi, E. Gülmez, M. Kaya, O. Kaya, Ö. Özçelik, S. Tekten, E. A. Yetkin, A. Cakir, K. Cankocak, Y. Komurcu, S. Sen, F. Aydogmus Sen, S. Cerci, B. Kaynak, S. Ozkorucuklu, D. Sunar Cerci, B. Grynyov, L. Levchuk, E. Bhal, S. Bologna, J. J. Brooke, E. Clement, D. Cussans, H. Flacher, J. Goldstein, G. P. Heath, H. F. Heath, L. Kreczko, B. Krikler, S. Paramesvaran, T. Sakuma, S. Seif El Nasr-Storey, V. J. Smith, J. Taylor, A. Titterton, K. W. Bell, A. Belyaev, C. Brew, R. M. Brown, D. J. A. Cockerill, K. V. Ellis, K. Harder, S. Harper, J. Linacre, K. Manolopoulos, D. M. Newbold, E. Olaiya, D. Petyt, T. Reis, T. Schuh, C. H. Shepherd-Themistocleous, A. Thea, I. R. Tomalin, T. Williams, R. Bainbridge, P. Bloch, S. Bonomally, J. Borg, S. Breeze, O. Buchmuller, A. Bundock, V. Cepaitis, G. S. Chahal, D. Colling, P. Dauncey, G. Davies, M. Della Negra, G. Fedi, G. Hall, G. Iles, J. Langford, L. Lyons, A.-M. Magnan, S. Malik, A. Martelli, V. Milosevic, J. Nash, V. Palladino, M. Pesaresi, D. M. Raymond, A. Richards, A. Rose, E. Scott, C. Seez, A. Shtipliyski, M. Stoye, A. Tapper, K. Uchida, T. Virdee, N. Wardle, S. N. Webb, D. Winterbottom, A. G. Zecchinelli, J. E. Cole, P. R. Hobson, A. Khan, P. Kyberd, C. K. Mackay, I. D. Reid, L. Teodorescu, S. Zahid, A. Brinkerhoff, K. Call, B. Caraway, J. Dittmann, K. Hatakeyama, A. R. Kanuganti, C. Madrid, B. McMaster, N. Pastika, S. Sawant, C. Smith, J. Wilson, R. Bartek, A. Dominguez, R. Uniyal, A. M. Vargas Hernandez, A. Buccilli, O. Charaf, S. I. Cooper, S. V. Gleyzer, C. Henderson, P. Rumerio, C. West, A. Akpinar, A. Albert, D. Arcaro, C. Cosby, Z. Demiragli, D. Gastler, J. Rohlf, K. Salyer, D. Sperka, D. Spitzbart, I. Suarez, S. Yuan, D. Zou, G. Benelli, B. Burkle, X. Coubez, D. Cutts, Y. t. Duh, M. Hadley, U. Heintz, J. M. Hogan, K. H. M. Kwok, E. Laird, G. Landsberg, K. T. Lau, J. Lee, M. Narain, S. Sagir, R. Syarif, E. Usai, W. Y. Wong, D. Yu, W. Zhang, R. Band, C. Brainerd, R. Breedon, M. Calderon De La Barca Sanchez, M. Chertok, J. Conway, R. Conway, P. T. Cox, R. Erbacher, C. Flores, G. Funk, F. Jensen, W. Ko, O. Kukral, R. Lander, M. Mulhearn, D. Pellett, J. Pilot, M. Shi, D. Taylor, K. Tos, M. Tripathi, Y. Yao, F. Zhang, M. Bachtis, R. Cousins, A. Dasgupta, D. Hamilton, J. Hauser, M. Ignatenko, T. Lam, N. Mccoll, W. A. Nash, S. Regnard, D. Saltzberg, C. Schnaible, B. Stone, V. Valuev, K. Burt, Y. Chen, R. Clare, J. W. Gary, S. M. A. Ghiasi Shirazi, G. Hanson, G. Karapostoli, O. R. Long, N. Manganelli, M. Olmedo Negrete, M. I. Paneva, W. Si, S. Wimpenny, Y. Zhang, J. G. Branson, P. Chang, S. Cittolin, S. Cooperstein, N. Deelen, J. Duarte, R. Gerosa, D. Gilbert, V. Krutelyov, J. Letts, M. Masciovecchio, S. May, S. Padhi, M. Pieri, V. Sharma, M. Tadel, F. Würthwein, A. Yagil, N. Amin, C. Campagnari, M. Citron, A. Dorsett, V. Dutta, J. Incandela, B. Marsh, H. Mei, A. Ovcharova, H. Qu, M. Quinnan, J. Richman, U. Sarica, D. Stuart, S. Wang, D. Anderson, A. Bornheim, O. Cerri, I. Dutta, J. M. Lawhorn, N. Lu, J. Mao, H. B. Newman, J. Ngadiuba, T. Q. Nguyen, J. Pata, M. Spiropulu, J. R. Vlimant, C. Wang, S. Xie, Z. Zhang, R. Y. Zhu, J. Alison, M. B. Andrews, T. Ferguson, T. Mudholkar, M. Paulini, M. Sun, I. Vorobiev, J. P. Cumalat, W. T. Ford, E. MacDonald, T. Mulholland, R. Patel, A. Perloff, K. Stenson, K. A. Ulmer, S. R. Wagner, J. Alexander, Y. Cheng, J. Chu, D. J. Cranshaw, A. Datta, A. Frankenthal, K. Mcdermott, J. Monroy, J. R. Patterson, D. Quach, A. Ryd, W. Sun, S. M. Tan, Z. Tao, J. Thom, P. Wittich, M. Zientek, S. Abdullin, M. Albrow, M. Alyari, G. Apollinari, A. Apresyan, A. Apyan, S. Banerjee, L. A. T. Bauerdick, A. Beretvas, D. Berry, J. Berryhill, P. C. Bhat, K. Burkett, J. N. Butler, A. Canepa, G. B. Cerati, H. W. K. Cheung, F. Chlebana, M. Cremonesi, V. D. Elvira, J. Freeman, Z. Gecse, E. Gottschalk, L. Gray, D. Green, S. Grünendahl, O. Gutsche, R. M. Harris, S. Hasegawa, R. Heller, T. C. Herwig, J. Hirschauer, B. Jayatilaka, S. Jindariani, M. Johnson, U. Joshi, P. Klabbers, T. Klijnsma, B. Klima, M. J. Kortelainen, S. Lammel, D. Lincoln, R. Lipton, M. Liu, T. Liu, J. Lykken, K. Maeshima, D. Mason, P. McBride, P. Merkel, S. Mrenna, S. Nahn, V. O’Dell, V. Papadimitriou, K. Pedro, C. Pena, O. Prokofyev, F. Ravera, A. Reinsvold Hall, L. Ristori, B. Schneider, E. Sexton-Kennedy, N. Smith, A. Soha, W. J. Spalding, L. Spiegel, S. Stoynev, J. Strait, L. Taylor, S. Tkaczyk, N. V. Tran, L. Uplegger, E. W. Vaandering, H. A. Weber, A. Woodard, D. Acosta, P. Avery, D. Bourilkov, L. Cadamuro, V. Cherepanov, F. Errico, R. D. Field, D. Guerrero, B. M. Joshi, M. Kim, J. Konigsberg, A. Korytov, K. H. Lo, K. Matchev, N. Menendez, G. Mitselmakher, D. Rosenzweig, K. Shi, J. Sturdy, J. Wang, S. Wang, X. Zuo, T. Adams, A. Askew, D. Diaz, R. Habibullah, S. Hagopian, V. Hagopian, K. F. Johnson, R. Khurana, T. Kolberg, G. Martinez, H. Prosper, C. Schiber, R. Yohay, J. Zhang, M. M. Baarmand, S. Butalla, T. Elkafrawy, M. Hohlmann, D. Noonan, M. Rahmani, M. Saunders, F. Yumiceva, M. R. Adams, L. Apanasevich, H. Becerril Gonzalez, R. Cavanaugh, X. Chen, S. Dittmer, O. Evdokimov, C. E. Gerber, D. A. Hangal, D. J. Hofman, C. Mills, G. Oh, T. Roy, M. B. Tonjes, N. Varelas, J. Viinikainen, X. Wang, Z. Wu, M. Alhusseini, K. Dilsiz, S. Durgut, R. P. Gandrajula, M. Haytmyradov, V. Khristenko, O. K. Köseyan, J.-P. Merlo, A. Mestvirishvili, A. Moeller, J. Nachtman, H. Ogul, Y. Onel, F. Ozok, A. Penzo, C. Snyder, E. Tiras, J. Wetzel, K. Yi, O. Amram, B. Blumenfeld, L. Corcodilos, M. Eminizer, A. V. Gritsan, S. Kyriacou, P. Maksimovic, C. Mantilla, J. Roskes, M. Swartz, T. Á. Vámi, C. Baldenegro Barrera, P. Baringer, A. Bean, A. Bylinkin, T. Isidori, S. Khalil, J. King, G. Krintiras, A. Kropivnitskaya, C. Lindsey, N. Minafra, M. Murray, C. Rogan, C. Royon, S. Sanders, E. Schmitz, J. D. Tapia Takaki, Q. Wang, J. Williams, G. Wilson, S. Duric, A. Ivanov, K. Kaadze, D. Kim, Y. Maravin, T. Mitchell, A. Modak, A. Mohammadi, F. Rebassoo, D. Wright, E. Adams, A. Baden, O. Baron, A. Belloni, S. C. Eno, Y. Feng, N. J. Hadley, S. Jabeen, G. Y. Jeng, R. G. Kellogg, T. Koeth, A. C. Mignerey, S. Nabili, M. Seidel, A. Skuja, S. C. Tonwar, L. Wang, K. Wong, D. Abercrombie, B. Allen, R. Bi, S. Brandt, W. Busza, I. A. Cali, Y. Chen, M. D’Alfonso, G. Gomez Ceballos, M. Goncharov, P. Harris, D. Hsu, M. Hu, M. Klute, D. Kovalskyi, J. Krupa, Y.-J. Lee, P. D. Luckey, B. Maier, A. C. Marini, C. Mcginn, C. Mironov, S. Narayanan, X. Niu, C. Paus, D. Rankin, C. Roland, G. Roland, Z. Shi, G. S. F. Stephans, K. Sumorok, K. Tatar, D. Velicanu, J. Wang, T. W. Wang, Z. Wang, B. Wyslouch, R. M. Chatterjee, A. Evans, S. Guts, P. Hansen, J. Hiltbrand, Sh. Jain, M. Krohn, Y. Kubota, Z. Lesko, J. Mans, M. Revering, R. Rusack, R. Saradhy, N. Schroeder, N. Strobbe, M. A. Wadud, J. G. Acosta, S. Oliveros, K. Bloom, S. Chauhan, D. R. Claes, C. Fangmeier, L. Finco, F. Golf, J. R. González Fernández, I. Kravchenko, J. E. Siado, G. R. Snow, B. Stieger, W. Tabb, F. Yan, G. Agarwal, H. Bandyopadhyay, C. Harrington, L. Hay, I. Iashvili, A. Kharchilava, C. McLean, D. Nguyen, J. Pekkanen, S. Rappoccio, B. Roozbahani, G. Alverson, E. Barberis, C. Freer, Y. Haddad, A. Hortiangtham, J. Li, G. Madigan, B. Marzocchi, D. M. Morse, V. Nguyen, T. Orimoto, A. Parker, L. Skinnari, A. Tishelman-Charny, T. Wamorkar, B. Wang, A. Wisecarver, D. Wood, S. Bhattacharya, J. Bueghly, Z. Chen, A. Gilbert, T. Gunter, K. A. Hahn, N. Odell, M. H. Schmitt, K. Sung, M. Velasco, R. Bucci, N. Dev, R. Goldouzian, M. Hildreth, K. Hurtado Anampa, C. Jessop, D. J. Karmgard, K. Lannon, N. Loukas, N. Marinelli, I. Mcalister, F. Meng, K. Mohrman, Y. Musienko, R. Ruchti, P. Siddireddy, S. Taroni, M. Wayne, A. Wightman, M. Wolf, L. Zygala, J. Alimena, B. Bylsma, B. Cardwell, L. S. Durkin, B. Francis, C. Hill, A. Lefeld, B. L. Winer, B. R. Yates, P. Das, G. Dezoort, P. Elmer, B. Greenberg, N. Haubrich, S. Higginbotham, A. Kalogeropoulos, G. Kopp, S. Kwan, D. Lange, M. T. Lucchini, J. Luo, D. Marlow, K. Mei, I. Ojalvo, J. Olsen, C. Palmer, P. Piroué, D. Stickland, C. Tully, S. Malik, S. Norberg, V. E. Barnes, R. Chawla, S. Das, L. Gutay, M. Jones, A. W. Jung, G. Negro, N. Neumeister, C. C. Peng, S. Piperov, A. Purohit, H. Qiu, J. F. Schulte, M. Stojanovic, N. Trevisani, F. Wang, R. Xiao, W. Xie, T. Cheng, J. Dolen, N. Parashar, A. Baty, S. Dildick, K. M. Ecklund, S. Freed, F. J. M. Geurts, M. Kilpatrick, A. Kumar, W. Li, B. P. Padley, R. Redjimi, J. Roberts, J. Rorie, W. Shi, A. G. Stahl Leiton, A. Bodek, P. de Barbaro, R. Demina, J. L. Dulemba, C. Fallon, T. Ferbel, M. Galanti, A. Garcia-Bellido, O. Hindrichs, A. Khukhunaishvili, E. Ranken, R. Taus, B. Chiarito, J. P. Chou, A. Gandrakota, Y. Gershtein, E. Halkiadakis, A. Hart, M. Heindl, E. Hughes, S. Kaplan, O. Karacheban, I. Laflotte, A. Lath, R. Montalvo, K. Nash, M. Osherson, S. Salur, S. Schnetzer, S. Somalwar, R. Stone, S. A. Thayil, S. Thomas, H. Wang, H. Acharya, A. G. Delannoy, S. Spanier, O. Bouhali, M. Dalchenko, A. Delgado, R. Eusebi, J. Gilmore, T. Huang, T. Kamon, H. Kim, S. Luo, S. Malhotra, R. Mueller, D. Overton, L. Perniè, D. Rathjens, A. Safonov, N. Akchurin, J. Damgov, V. Hegde, S. Kunori, K. Lamichhane, S. W. Lee, T. Mengke, S. Muthumuni, T. Peltola, S. Undleeb, I. Volobouev, Z. Wang, A. Whitbeck, E. Appelt, S. Greene, A. Gurrola, R. Janjam, W. Johns, C. Maguire, A. Melo, H. Ni, K. Padeken, F. Romeo, P. Sheldon, S. Tuo, J. Velkovska, M. Verweij, M. W. Arenton, B. Cox, G. Cummings, J. Hakala, R. Hirosky, M. Joyce, A. Ledovskoy, A. Li, C. Neu, B. Tannenwald, Y. Wang, E. Wolfe, F. Xia, P. E. Karchin, N. Poudyal, P. Thapa, K. Black, T. Bose, J. Buchanan, C. Caillol, S. Dasu, I. De Bruyn, P. Everaerts, C. Galloni, H. He, M. Herndon, A. Hervé, U. Hussain, A. Lanaro, A. Loeliger, R. Loveless, J. Madhusudanan Sreekala, A. Mallampalli, D. Pinna, T. Ruggles, A. Savin, V. Shang, V. Sharma, W. H. Smith, J. Steggemann, D. Teague, S. Trembath-Reichert, W. Vetens, CMS Collaboration

**Affiliations:** 1 Yerevan Physics Institute, Yerevan, Armenia; 2 Institut für Hochenergiephysik, Wien, Austria; 3 Institute for Nuclear Problems, Minsk, Belarus; 4 Universiteit Antwerpen, Antwerp, Belgium; 5 Vrije Universiteit Brussel, Brussel, Belgium; 6 Université Libre de Bruxelles, Bruxelles, Belgium; 7 Ghent University, Ghent, Belgium; 8 Université Catholique de Louvain, Louvain-la-Neuve, Belgium; 9 Centro Brasileiro de Pesquisas Fisicas, Rio de Janeiro, Brazil; 10 Universidade do Estado do Rio de Janeiro, Rio de Janeiro, Brazil; 11 Universidade Estadual Paulista, Universidade Federal do ABC, São Paulo, Brazil; 12 Institute for Nuclear Research and Nuclear Energy, Bulgarian Academy of Sciences, Sofia, Bulgaria; 13 University of Sofia, Sofia, Bulgaria; 14 Beihang University, Beijing, China; 15 Department of Physics, Tsinghua University, Beijing, China; 16 Institute of High Energy Physics, Beijing, China; 17 State Key Laboratory of Nuclear Physics and Technology, Peking University, Beijing, China; 18 Sun Yat-Sen University, Guangzhou, China; 19 Institute of Modern Physics and Key Laboratory of Nuclear Physics and Ion-beam Application (MOE), Fudan University, Shanghai, China; 20 Zhejiang University, Hangzhou, China; 21 Universidad de Los Andes, Bogota, Colombia; 22 Universidad de Antioquia, Medellin, Colombia; 23 University of Split, Faculty of Electrical Engineering, Mechanical Engineering and Naval Architecture, Split, Croatia; 24 University of Split, Faculty of Science, Split, Croatia; 25 Institute Rudjer Boskovic, Zagreb, Croatia; 26 University of Cyprus, Nicosia, Cyprus; 27 Charles University, Prague, Czech Republic; 28 Escuela Politecnica Nacional, Quito, Ecuador; 29 Universidad San Francisco de Quito, Quito, Ecuador; 30 Academy of Scientific Research and Technology of the Arab Republic of Egypt, Egyptian Network of High Energy Physics, Cairo, Egypt; 31 Center for High Energy Physics (CHEP-FU), Fayoum University, El-Fayoum, Egypt; 32 National Institute of Chemical Physics and Biophysics, Tallinn, Estonia; 33 Department of Physics, University of Helsinki, Helsinki, Finland; 34 Helsinki Institute of Physics, Helsinki, Finland; 35 Lappeenranta University of Technology, Lappeenranta, Finland; 36 IRFU, CEA, Université Paris-Saclay, Gif-sur-Yvette, France; 37 Laboratoire Leprince-Ringuet, CNRS/IN2P3, Ecole Polytechnique, Institut Polytechnique de Paris, Palaiseau, France; 38 Université de Strasbourg, CNRS, IPHC UMR 7178, Strasbourg, France; 39 Institut de Physique des 2 Infinis de Lyon (IP2I ), Villeurbanne, France; 40 Georgian Technical University, Tbilisi, Georgia; 41 RWTH Aachen University, I. Physikalisches Institut, Aachen, Germany; 42 III. Physikalisches Institut A, RWTH Aachen University, Aachen, Germany; 43 III. Physikalisches Institut B, RWTH Aachen University, Aachen, Germany; 44 Deutsches Elektronen-Synchrotron, Hamburg, Germany; 45 University of Hamburg, Hamburg, Germany; 46 Karlsruher Institut fuer Technologie, Karlsruhe, Germany; 47 Institute of Nuclear and Particle Physics (INPP), NCSR Demokritos, Aghia Paraskevi, Athens, Greece; 48 National and Kapodistrian University of Athens, Athens, Greece; 49 National Technical University of Athens, Athens, Greece; 50 University of Ioánnina, Ioannina, Greece; 51 MTA-ELTE Lendület CMS Particle and Nuclear Physics Group, Eötvös Loránd University, Budapest, Hungary; 52 Wigner Research Centre for Physics, Budapest, Hungary; 53 Institute of Nuclear Research ATOMKI, Debrecen, Hungary; 54 Institute of Physics, University of Debrecen, Debrecen, Hungary; 55 Eszterhazy Karoly University, Karoly Robert Campus, Gyongyos, Hungary; 56 Indian Institute of Science (IISc), Bangalore, India; 57 National Institute of Science Education and Research, HBNI, Bhubaneswar, India; 58 Panjab University, Chandigarh, India; 59 University of Delhi, Delhi, India; 60 Saha Institute of Nuclear Physics, HBNI, Kolkata, India; 61 Indian Institute of Technology Madras, Madras, India; 62 Bhabha Atomic Research Centre, Mumbai, India; 63 Tata Institute of Fundamental Research-A, Mumbai, India; 64 Tata Institute of Fundamental Research-B, Mumbai, India; 65 Indian Institute of Science Education and Research (IISER), Pune, India; 66 Department of Physics, Isfahan University of Technology, Isfahan, Iran; 67 Institute for Research in Fundamental Sciences (IPM), Tehran, Iran; 68 University College Dublin, Dublin, Ireland; 69 INFN Sezione di Bari , Universit’a di Bari, Politecnico di Bari, Bari, Italy; 70 INFN Sezione di Bologna, Università di Bologna, Bologna, Italy; 71 INFN Sezione di Catania, Università di Catania, Catania, Italy; 72 INFN Sezione di Firenze, Università di Firenze, Firenze, Italy; 73 INFN Laboratori Nazionali di Frascati, Frascati, Italy; 74 INFN Sezione di Genova, Università di Genova, Genoa, Italy; 75 INFN Sezione di Milano-Bicocca, Università di Milano-Bicocca, Milan, Italy; 76 INFN Sezione di Napoli , Università di Napoli ’Federico II’ , Napoli, Italy, Università della Basilicata , Potenza, Italy, Università G. Marconi, Rome, Italy; 77 INFN Sezione di Padova , Università di Padova , Padova, Italy, Università di Trento, Trento, Italy; 78 INFN Sezione di Pavia, Università di Pavia, Pavia, Italy; 79 INFN Sezione di Perugia, Università di Perugia, Perugia, Italy; 80 INFN Sezione di Pisa , Università di Pisa , Scuola Normale Superiore di Pisa , Pisa Italy, Università di Siena, Siena, Italy; 81 INFN Sezione di Roma, Sapienza Università di Roma, Rome, Italy; 82 INFN Sezione di Torino , Università di Torino , Torino Torino, Italy, Università del Piemonte Orientale, Novara, Italy; 83 INFN Sezione di Trieste, Università di Trieste, Trieste, Italy; 84 Kyungpook National University, Daegu, Korea; 85 Chonnam National University, Institute for Universe and Elementary Particles, Kwangju, Korea; 86 Hanyang University, Seoul, Korea; 87 Korea University, Seoul, Korea; 88 Kyung Hee University, Department of Physics, Seoul, Republic of Korea; 89 Sejong University, Seoul, Korea; 90 Seoul National University, Seoul, Korea; 91 University of Seoul, Seoul, Korea; 92 Department of Physics, Yonsei University, Seoul, Korea; 93 Sungkyunkwan University, Suwon, Korea; 94 Riga Technical University, Riga, Latvia; 95 Vilnius University, Vilnius, Lithuania; 96 National Centre for Particle Physics, Universiti Malaya, Kuala Lumpur, Malaysia; 97 Universidad de Sonora (UNISON), Hermosillo, Mexico; 98 Centro de Investigacion y de Estudios Avanzados del IPN, Mexico City, Mexico; 99 Universidad Iberoamericana, Mexico City, Mexico; 100 Benemerita Universidad Autonoma de Puebla, Puebla, Mexico; 101 Universidad Autónoma de San Luis Potosí, San Luis Potosí, Mexico; 102 University of Montenegro, Podgorica, Montenegro; 103 University of Auckland, Auckland, New Zealand; 104 University of Canterbury, Christchurch, New Zealand; 105 National Centre for Physics, Quaid-I-Azam University, Islamabad, Pakistan; 106 AGH University of Science and Technology Faculty of Computer Science, Electronics and Telecommunications, Kraków, Poland; 107 National Centre for Nuclear Research, Swierk, Poland; 108 Institute of Experimental Physics, Faculty of Physics, University of Warsaw, Warsaw, Poland; 109 Laboratório de Instrumentação e Física Experimental de Partículas, Lisbon, Portugal; 110 Joint Institute for Nuclear Research, Dubna, Russia; 111 Petersburg Nuclear Physics Institute, Gatchina (St. Petersburg), Russia; 112 Institute for Nuclear Research, Moscow, Russia; 113 Institute for Theoretical and Experimental Physics named by A.I. Alikhanov of NRC ‘Kurchatov Institute’, Moscow, Russia; 114 Moscow Institute of Physics and Technology, Moscow, Russia; 115 National Research Nuclear University ‘Moscow Engineering Physics Institute’ (MEPhI), Moscow, Russia; 116 P.N. Lebedev Physical Institute, Moscow, Russia; 117 Skobeltsyn Institute of Nuclear Physics, Lomonosov Moscow State University, Moscow, Russia; 118 Novosibirsk State University (NSU), Novosibirsk, Russia; 119 Institute for High Energy Physics of National Research Centre ‘Kurchatov Institute’, Protvino, Russia; 120 National Research Tomsk Polytechnic University, Tomsk, Russia; 121 Tomsk State University, Tomsk, Russia; 122 University of Belgrade: Faculty of Physics and VINCA Institute of Nuclear Sciences, Belgrade, Serbia; 123 Centro de Investigaciones Energéticas Medioambientales y Tecnológicas (CIEMAT), Madrid, Spain; 124 Universidad Autónoma de Madrid, Madrid, Spain; 125 Universidad de Oviedo, Instituto Universitario de Ciencias y Tecnologías Espaciales de Asturias (ICTEA), Oviedo, Spain; 126 Instituto de Física de Cantabria (IFCA), CSIC-Universidad de Cantabria, Santander, Spain; 127 University of Colombo, Colombo, Sri Lanka; 128 Department of Physics, University of Ruhuna, Matara, Sri Lanka; 129 CERN, European Organization for Nuclear Research, Geneva, Switzerland; 130 Paul Scherrer Institut, Villigen, Switzerland; 131 ETH Zurich-Institute for Particle Physics and Astrophysics (IPA), Zurich, Switzerland; 132 Universität Zürich, Zurich, Switzerland; 133 National Central University, Chung-Li, Taiwan; 134 National Taiwan University (NTU), Taipei, Taiwan; 135 Department of Physics, Faculty of Science, Chulalongkorn University, Bangkok, Thailand; 136 Physics Department, Science and Art Faculty, Çukurova University, Adana, Turkey; 137 Physics Department, Middle East Technical University, Ankara, Turkey; 138 Bogazici University, Istanbul, Turkey; 139 Istanbul Technical University, Istanbul, Turkey; 140 Istanbul University, Istanbul, Turkey; 141 Institute for Scintillation Materials of National Academy of Science of Ukraine, Kharkov, Ukraine; 142 National Scientific Center, Kharkov Institute of Physics and Technology, Kharkov, Ukraine; 143 University of Bristol, Bristol, UK; 144 Rutherford Appleton Laboratory, Didcot, UK; 145 Imperial College, London, UK; 146 Brunel University, Uxbridge, UK; 147 Baylor University, Waco, USA; 148 Catholic University of America, Washington, DC USA; 149 The University of Alabama, Tuscaloosa, USA; 150 Boston University, Boston, USA; 151 Brown University, Providence, USA; 152 University of California, Davis, Davis, USA; 153 University of California, Los Angeles, USA; 154 University of California, Riverside, Riverside, USA; 155 University of California, San Diego, La Jolla, USA; 156 Department of Physics, University of California, Santa Barbara, Santa Barbara, USA; 157 California Institute of Technology, Pasadena, USA; 158 Carnegie Mellon University, Pittsburgh, USA; 159 University of Colorado Boulder, Boulder, USA; 160 Cornell University, Ithaca, USA; 161 Fermi National Accelerator Laboratory, Batavia, USA; 162 University of Florida, Gainesville, USA; 163 Florida State University, Tallahassee, USA; 164 Florida Institute of Technology, Melbourne, USA; 165 University of Illinois at Chicago (UIC), Chicago, USA; 166 The University of Iowa, Iowa City, USA; 167 Johns Hopkins University, Baltimore, USA; 168 The University of Kansas, Lawrence, USA; 169 Kansas State University, Manhattan, USA; 170 Lawrence Livermore National Laboratory, Livermore, USA; 171 University of Maryland, College Park, USA; 172 Massachusetts Institute of Technology, Cambridge, USA; 173 University of Minnesota, Minneapolis, USA; 174 University of Mississippi, Oxford, USA; 175 University of Nebraska-Lincoln, Lincoln, USA; 176 State University of New York at Buffalo, Buffalo, USA; 177 Northeastern University, Boston, USA; 178 Northwestern University, Evanston, USA; 179 University of Notre Dame, Notre Dame, USA; 180 The Ohio State University, Columbus, USA; 181 Princeton University, Princeton, USA; 182 University of Puerto Rico, Mayaguez, USA; 183 Purdue University, West Lafayette, USA; 184 Purdue University Northwest, Hammond, USA; 185 Rice University, Houston, USA; 186 University of Rochester, Rochester, USA; 187 Rutgers, The State University of New Jersey, Piscataway, USA; 188 University of Tennessee, Knoxville, USA; 189 Texas A&M University, College Station, USA; 190 Texas Tech University, Lubbock, USA; 191 Vanderbilt University, Nashville, USA; 192 University of Virginia, Charlottesville, USA; 193 Wayne State University, Detroit, USA; 194 University of Wisconsin-Madison, Madison, WI USA; 195 Vienna University of Technology, Vienna, Austria; 196 Institute of Basic and Applied Sciences, Faculty of Engineering, Arab Academy for Science, Technology and Maritime Transport, Alexandria, Egypt; 197 Université Libre de Bruxelles, Bruxelles, Belgium; 198 IRFU, CEA, Université Paris-Saclay, Gif-sur-Yvette, France; 199 Universidade Estadual de Campinas, Campinas, Brazil; 200 Federal University of Rio Grande do Sul, Porto Alegre, Brazil; 201 UFMS, Nova Andradina, Brazil; 202 Universidade Federal de Pelotas, Pelotas, Brazil; 203 University of Chinese Academy of Sciences, Beijing, China; 204 Institute for Theoretical and Experimental Physics named by A.I. Alikhanov of NRC ‘Kurchatov Institute’, Moscow, Russia; 205 Joint Institute for Nuclear Research, Dubna, Russia; 206 Cairo University, Cairo, Egypt; 207 Helwan University, Cairo, Egypt; 208 Zewail City of Science and Technology, Zewail, Egypt; 209 British University in Egypt, Cairo, Egypt; 210 Fayoum University, El-Fayoum, Egypt; 211 Purdue University, West Lafayette, USA; 212 Université de Haute Alsace, Mulhouse, France; 213 Erzincan Binali Yildirim University, Erzincan, Turkey; 214 CERN, European Organization for Nuclear Research, Geneva, Switzerland; 215 III. Physikalisches Institut A, RWTH Aachen University, Aachen, Germany; 216 University of Hamburg, Hamburg, Germany; 217 Department of Physics, Isfahan University of Technology, Isfahan, Iran; 218 Brandenburg University of Technology, Cottbus, Germany; 219 Skobeltsyn Institute of Nuclear Physics, Lomonosov Moscow State University, Moscow, Russia; 220 Institute of Physics, University of Debrecen, Debrecen, Hungary; 221 Physics Department, Faculty of Science, Assiut University, Assiut, Egypt; 222 MTA-ELTE Lendület CMS Particle and Nuclear Physics Group, Eötvös Loránd University, Budapest, Hungary; 223 Institute of Nuclear Research ATOMKI, Debrecen, Hungary; 224 IIT Bhubaneswar, Bhubaneswar, India; 225 Institute of Physics, Bhubaneswar, India; 226 G.H.G. Khalsa College, Punjab, India; 227 Shoolini University, Solan, India; 228 University of Hyderabad, Hyderabad, India; 229 University of Visva-Bharati, Santiniketan, India; 230 Indian Institute of Technology (IIT), Mumbai, India; 231 Deutsches Elektronen-Synchrotron, Hamburg, Germany; 232 Sharif University of Technology, Tehran, Iran; 233 Department of Physics, University of Science and Technology of Mazandaran, Behshahr, Iran; 234 INFN Sezione di Bari , Università di Bari, Politecnico di Bari, Bari, Italy; 235 Italian National Agency for New Technologies, Energy and Sustainable Economic Development, Bologna, Italy; 236 Centro Siciliano di Fisica Nucleare e di Struttura Della Materia, Catania, Italy; 237 Università di Napoli ’Federico II’, Naples, Italy; 238 Riga Technical University, Riga, Latvia; 239 Consejo Nacional de Ciencia y Tecnología, Mexico City, Mexico; 240 Warsaw University of Technology, Institute of Electronic Systems, Warsaw, Poland; 241 Institute for Nuclear Research, Moscow, Russia; 242 National Research Nuclear University ‘Moscow Engineering Physics Institute’ (MEPhI), Moscow, Russia; 243 St. Petersburg State Polytechnical University, St. Petersburg, Russia; 244 University of Florida, Gainesville, USA; 245 Imperial College, London, UK; 246 P.N. Lebedev Physical Institute, Moscow, Russia; 247 California Institute of Technology, Pasadena, USA; 248 Budker Institute of Nuclear Physics, Novosibirsk, Russia; 249 Faculty of Physics, University of Belgrade, Belgrade, Serbia; 250 Trincomalee Campus, Eastern University, Nilaveli, Sri Lanka; 251 INFN Sezione di Pavia, Università di Pavia, Pavia, Italy; 252 National and Kapodistrian University of Athens, Athens, Greece; 253 Universität Zürich, Zurich, Switzerland; 254 Stefan Meyer Institute for Subatomic Physics, Vienna, Austria; 255 Laboratoire d’Annecy-le-Vieux de Physique des Particules, IN2P3-CNRS, Annecy-le-Vieux, France; 256 Şırnak University, Sirnak, Turkey; 257 Department of Physics, Tsinghua University, Beijing, China; 258 Near East University, Research Center of Experimental Health Science, Nicosia, Turkey; 259 Beykent University, Istanbul, Turkey; 260 Application and Research Center for Advanced Studies (App. & Res. Cent. for Advanced Studies), Istanbul Aydin University, Istanbul, Turkey; 261 Mersin University, Mersin, Turkey; 262 Piri Reis University, Istanbul, Turkey; 263 Adiyaman University, Adiyaman, Turkey; 264 Ozyegin University, Istanbul, Turkey; 265 Izmir Institute of Technology, Izmir, Turkey; 266 Necmettin Erbakan University, Konya, Turkey; 267 Bozok Universitetesi Rektörlügü, Yozgat, Turkey; 268 Marmara University, Istanbul, Turkey; 269 Milli Savunma University, Istanbul, Turkey; 270 Kafkas University, Kars, Turkey; 271 Istanbul Bilgi University, Istanbul, Turkey; 272 Hacettepe University, Ankara, Turkey; 273 School of Physics and Astronomy, University of Southampton, Southampton, UK; 274 IPPP Durham University, Durham, UK; 275 Monash University, Faculty of Science, Clayton, Australia; 276 Bethel University, St. Paul, Minneapolis, USA; 277 Karamanoğlu Mehmetbey University, Karaman, Turkey; 278 Ain Shams University, Cairo, Egypt; 279 Bingol University, Bingol, Turkey; 280 Georgian Technical University, Tbilisi, Georgia; 281 Sinop University, Sinop, Turkey; 282 Mimar Sinan University, Istanbul, Turkey; 283 Department of Physics, Nanjing Normal University, Nanjing, China; 284 Texas A&M University at Qatar, Doha, Qatar; 285 Kyungpook National University, Daegu, Korea; 286 CERN, 1211 Geneva 23, Switzerland

## Abstract

Collinear (small-angle) and large-angle, as well as soft and hard radiations are investigated in three-jet and ${\text {Z}}$ + two-jet events collected in proton-proton collisions at the LHC. The normalized production cross sections are measured as a function of the ratio of transverse momenta of two jets and their angular separation. The measurements in the three-jet and ${\text {Z}}$ + two-jet events are based on data collected at a center-of-mass energy of 8$\,{\text {TeV}}$, corresponding to an integrated luminosity of 19.8$\,\text {fb}^{-1}$. The ${\text {Z}}$ + two-jet events are reconstructed in the dimuon decay channel of the ${\text {Z}}$ boson. The three-jet measurement is extended to include $\sqrt{s} = 13\,{\text {TeV}}$ data corresponding to an integrated luminosity of 2.3$\,\text {fb}^{-1}$. The results are compared to predictions from event generators that include parton showers, multiple parton interactions, and hadronization. The collinear and soft regions are in general well described by parton showers, whereas the regions of large angular separation are often best described by calculations using higher-order matrix elements.

## Introduction

Collimated streams of particles, produced in interactions of quarks and gluons and reconstructed as jets, are described by the theory of strong interactions, quantum chromodynamics (QCD). Multijet events provide exemplary signatures in high-energy collider experiments, and modeling their characteristics plays an important role in precision measurements, as well as in searches for new physics. The understanding of the structure of multijet final states is therefore crucial for analyses of those events.

Theoretical predictions for multijet events are based on a matrix element (ME) expansion to a fixed perturbative order, supplemented by the parton shower (PS) approach to approximate higher-order perturbative contributions. The ME expansion incorporates color correlations between quarks and gluons, including interference terms, as well as kinematic correlations between the partons, without any approximation at fixed perturbative order. Its application is, however, currently limited to final states with less than ${\mathcal {O}}(10)$ partons. The PS can simulate final states containing many partons, but with probabilities calculated using the approximations of soft and collinear kinematics and partial or averaged color structures. The best descriptions of multijet final states are based on a combination of both approaches [1–4]. Other features implemented in simulations, such as multiple parton interactions (MPI) and hadronization, also play an important role, e.g., in describing angular correlations between jets [5–7].

**Fig. 1:**
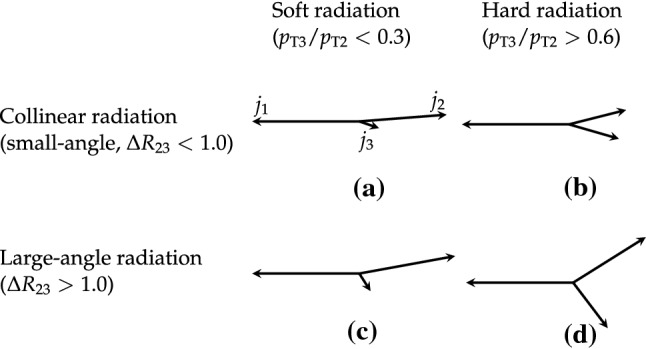
Four categories of parton radiation. **a** Soft and small-angle radiation, **b** hard and small-angle radiation, **c** soft and large-angle radiation, **d** hard and large-angle radiation

In this paper, we investigate collinear (small-angle) and large-angle radiation in different regions of jet transverse momentum ($p_{\mathrm {T}}$) by concentrating on two different topologies, one using three-jet events and another with ${\text {Z}}$ + two-jet events. We label the hardest jet, or ${\text {Z}}$ boson as $j_1$, the next hardest as $j_2$, and the softest as $j_3$. We introduce two observables that are sensitive to the dynamic properties of multijet final states. One observable is the $p_{\mathrm {T}}$ ratio of $j_3$ to $j_2$, $p_{\mathrm {T3}}/p_{\mathrm {T2}}$. The other observable is the angular distance between the jet centers of $j_2$ and $j_3$ in the rapidity-azimuth (*y*-$\phi$) phase space, $\varDelta R_{23} = \sqrt{\smash [b]{(y_{3} -y_{2})^{2} + (\phi _{3} - \phi _{2})^{2}}}$. The definition of rapidity is $y = \ln \sqrt{(E+p_{z}c)/(E-p_{z}c)}$, and the definitions of other kinematic variables are given in Ref. [8]. As indicated in Fig. 1, we classify three-jet and ${\text {Z}}$ + two-jet events into different categories using these two observables:

(i) soft ($p_{\mathrm {T3}}/p_{\mathrm {T2}} < 0.3$) or hard ($p_{\mathrm {T3}}/p_{\mathrm {T2}} > 0.6$) radiation, depending on the ratio $p_{\mathrm {T3}}/p_{\mathrm {T2}}$;
(ii) small-angle ($\varDelta R_{23} < 1.0$) or large-angle ($\varDelta R_{23} > 1.0$) radiation, depending on the angular separation $\varDelta R_{23}$.

According to these classifications, events in the soft and small-angle radiation region, as shown in Fig. 1a, can only be described if soft gluon resummation, e.g., in form of a parton shower, is included, whereas events in the hard and large-angle radiation region, as shown in Fig. 1d, would be better described when including the ME calculations. The events in Fig. 1b and c are also of interest, since they should include effects from both the PS and ME.

We report on proton-proton (${\text {p}}{\text {p}}$) collision data collected at the CMS experiment containing three-jet events at center-of-mass energies of 8 and 13$\,{\text {TeV}}$, and ${\text {Z}}$ + two-jet events at a center-of-mass energy of 8$\,{\text {TeV}}$. The measurements are compared to calculations based on a leading-order (LO) or next-to-leading-order (NLO) ME supplemented with effects from PS, MPI, and hadronization. The NLO ME descriptions apply to the lowest parton multiplicities relevant to the selected events: 2 jets for the three-jet analysis and ${\text {Z}}$+1j  for the ${\text {Z}}$ + two-jet analysis. The measurements using three-jet final states are complementary to those with ${\text {Z}}$ + two-jet events in a sense that different kinematic regions and initial-state flavor compositions are being probed. The jets are also fully color connected, while the ${\text {Z}}$ boson is color neutral, so color coherence effects should not appear so strongly in ${\text {Z}}$ + two-jet events.

The goal of the measurements is: (i) to untangle the different features of the radiation in the collinear and large-angle events; (ii) to investigate how well the PS approach describes the hard and large-angle radiation patterns; and (iii) to illustrate how ME calculations can attempt to describe the soft and collinear regions.

## The CMS detector

The central feature of the CMS detector is a superconducting solenoid of 6$\,{\text {m}}$ internal diameter, providing a magnetic field of 3.8$\,{\text {T}}$. A silicon pixel and strip tracker, a lead tungstate crystal electromagnetic calorimeter (ECAL), and a brass and scintillator hadron calorimeter (HCAL), each composed of a barrel and two endcap sections, reside within the volume of the solenoid. Charged-particle trajectories are measured in the tracker with full azimuthal acceptance within pseudorapidities $|\eta | < 2.5$. The ECAL, which is equipped with a preshower detector in the endcaps, and the HCAL cover the region $|\eta | < 3.0$. Forward calorimeters extend the pseudorapidity coverage provided by the barrel and endcap detectors to the region $3.0<|\eta | < 5.2$. Finally, muons are measured up to $|\eta | < 2.4$ in gas-ionization detectors embedded in the steel flux-return yoke outside the solenoid. Events of interest are selected using a two-tiered trigger system [9]. The first level, composed of custom hardware processors, uses information from the calorimeters and muon detectors to select events at a rate of around 100$\,{\text {kHz}}$ within a fixed latency of about 4$\,\mu \text {s}$. The second level, known as the high-level trigger (HLT), consists of a farm of processors running a version of the full event reconstruction software optimized for fast processing, and reduces the event rate to around 1$\,{\text {kHz}}$ before data storage.

A more detailed description of the CMS detector, together with a definition of the coordinate system and the kinematic variables, is given in Ref. [8].

## Event samples and selection

The data in this study were collected with the CMS detector at the LHC using pp collisions at center-of-mass energies of 8 and 13$\,{\text {TeV}}$. The $\sqrt{s} = 8\,{\text {TeV}}$ data, taken in 2012 during LHC Run 1, correspond to an integrated luminosity of 19.8$\,\text {fb}^{-1}$, and the $\sqrt{s} = 13\,{\text {TeV}}$ data, taken in 2015 during LHC Run 2, correspond to an integrated luminosity of 2.3$\,\text {fb}^{-1}$.

Particles are reconstructed and identified using a particle-flow (PF) algorithm [10], that utilizes an optimized combination of information from the various elements of the CMS detector. Jets are reconstructed by clustering the four-vectors of the PF candidates with the infrared and collinear-safe anti-$k_{\mathrm {T}}$ clustering algorithm [11] using a distance parameter $R_{\mathrm {jet}} =$ 0.5 (0.4) at $\sqrt{s} = 8\,(13)\,{\text {TeV}}$. The clustering is performed with the FastJet software package [12]. The jets are ordered in $p_{\mathrm {T}}$ and all events with additional jets are analyzed. In addition, three-jet events use the charged-hadron subtraction (CHS) technique [10] to mitigate the effect of extraneous ${\text {p}}{\text {p}}$ collisions in the same bunch crossing (pileup, PU). The CHS technique reduces the contribution to the reconstructed jets from PU by removing tracks identified as originating from PU vertices.

Muons are reconstructed using a simultaneous global fit performed with the hits in the silicon tracker and the muon system. They are required to pass standard identification criteria [13, 14] based on the minimum number of hits in each detector, quality of the fit, and the consistency with the primary vertex by requiring the longitudinal (transverse) impact parameters to be less than 0.5 (0.2) $\,\text {cm}$. The efficiency to reconstruct and identify muons is greater than 95% over the entire region of pseudorapidity covered by the CMS muon system ($|\eta | > 2.4$). The overall momentum scale is measured to a precision of 0.2% with muons from ${\text {Z}}$ decays. The transverse momentum resolution varies from 1 to 6% depending on pseudorapidity for muons with $p_{\mathrm {T}}$ for a few $\,\text {GeV}$ to 100$\,\text {GeV}$ and reaches 10% for 1$\,{\text {TeV}}$ muons [15]. Observed distributions for muons are well reproduced by Monte Carlo (MC) simulation. Corresponding scale factors for the difference between data and MC simulations are measured with good accuracy [16]. Muons must be isolated from other activity in the tracker by requiring the $p_{\mathrm {T}}$ sum of other tracks within a cone of radius $\varDelta R = \sqrt{(\varDelta \eta )^{2} + (\varDelta \phi )^{2}} = 0.3$ centered on the muon candidate, is less than 10% of the muon $p_{\mathrm {T}}$. If the two muons with the highest $p_{\mathrm {T}}$ in an event are within the isolation cone of one another, the other muon candidate is removed from the isolation sum of each muon.

Three-jet events are collected using single jet HLT requirements that are not pre-scaled. The $\sqrt{s} = 8\,(13)\,{\text {TeV}}$ data use a 320 (450)$\,\text {GeV}$ trigger $p_{\mathrm {T}}$ threshold. In the offline analyses, the $p_{\mathrm {T}}$ threshold starts at 510$\,\text {GeV}$ for both sets of data. The ${\text {Z}}$ + two-jet events with the ${\text {Z}}$ boson decaying into a pair of muons are collected at $\sqrt{s} = 8\,{\text {TeV}}$ with a single-muon HLT that requires a muon $p_{\mathrm {T}} > 24\,\text {GeV}$ and $|\eta | < 2.1$.

In the three-jet systems, the leading jet is required to have a $p_{\mathrm {T}} > 510\,\text {GeV}$, because of a decreasing efficiency for single jet triggers below this value [9, 17, 18]. Events with at least three jets of $p_{\mathrm {T}} > 30\,\text {GeV}$ are selected for further consideration. The leading and subleading jets must be within a rapidity range of $|y | < 2.5$, and the third jet is therefore implicitly restricted to $|y | < 4$ by requiring $\varDelta R_{23} < 1.5$. A dijet topology with an extra jet is selected by requiring the difference in azimuthal angle between the first and second jet to be $\pi -1< \varDelta \phi _{12} < \pi$. The missing transverse momentum vector ${\mathbf {p}}_{\mathrm {T}}^{\text {miss}}$ is defined as the projection onto the plane perpendicular to the beam axis of the negative vector sum of the momentum of all reconstructed PF objects in an event. Its magnitude is referred to as $p_{\mathrm {T}} ^\text {miss}$. Events with a $p_{\mathrm {T}} ^\text {miss}$ divided by the scalar sum of all transverse momenta $> 0.3$ are rejected to remove the contamination from ${\text {W}}$or ${\text {Z}}$ boson decays [19–21]. To avoid an overlap between $j_2$ and $j_3$, $\varDelta R_{23}$ is required to be larger than the distance parameter $R_{\mathrm {jet}}$. We thus require $\varDelta R_{23}$ to be larger than 0.6 (0.5) for $\sqrt{s} = 8\,(13)\,{\text {TeV}}$ data. The maximum $\varDelta R_{23}$ is set to 1.5 to ensure that $j_3$ is closer to $j_2$ than to $j_1$. We further require that $0.1<p_{\mathrm {T3}}/p_{\mathrm {T2}}\ < 0.9$ to avoid $p_{\mathrm {T3}}$ threshold effects and to ensure $p_{\mathrm {T}}$ ordering for hard radiation.

In ${\text {Z}}$ + two-jet events, the ${\text {Z}}$ boson is reconstructed from a pair of oppositely charged, isolated muons with $p_{\mathrm {T}} > 25~(5)\,\text {GeV}$ and $|y | < 2.1$ (2.4) for the leading (subleading) muon. Muons are required to be from primary vertex with distance $dr < 0.2 \,\text {cm}$ and $dz < 0.5 \,\text {cm}$. The dimuon invariant mass is required to be $70< m_{\mu ^+\mu ^-} < 110\,\text {GeV}$ with the dimuon momentum satisfying $p_{\mathrm {T1}} > 80\,\text {GeV}$ and $|y_1 | < 2$. At least two jets are required in the final state with the leading jet (labeled $j_2$) satisfying $p_{\mathrm {T2}} > 80\,\text {GeV}$ and $|y_{2} | < 1$ and the subleading jet (labeled $j_3$) required to have $p_{\mathrm {T3}} > 20\,\text {GeV}$ with $|y_{3} | < 2.4$. The distance between muons from ${\text {Z}}$ bosons and jets are requested to be more then 0.5. The ${\text {Z}}$ + two-jet topology is further restricted by requiring a difference in the azimuthal angle between the ${\text {Z}}$ boson and $j_{2}$ of $\varDelta \phi _{12} > 2$.

Table 1 shows a summary of the event selection requirements for both samples.

**Table 1:** Phase space selection for the three-jet and ${\text {Z}}$ + two-jet analyses

Three-jet events	
Transverse momentum of the leading jet ($j_1$)	$p_{\mathrm {T1}} > 510\,\text {GeV}$
Transverse momentum of each jet and rapidity of $$j_{1,2}$$	$$p_{\mathrm {T}} > 30\,\text {GeV} $$ , $$|y_{1,2} | < 2.5 $$
Azimuthal angle difference between $j_1$ and $j_2$	$\pi -1< \varDelta \phi _{12} < \pi$
Transverse momentum ratio between $j_2$ and $j_3$	$0.1<p_{\mathrm {T3}}/p_{\mathrm {T2}} < 0.9$
Angular distance between $j_2$ and $j_3$	$R_{\mathrm {jet}}+0.1< \varDelta R_{23} < 1.5$
Number of selected events at $\sqrt{s} = 8\,(13) \,{\text {TeV}}$	777 618 (613 254)

${\text {Z}}$ + two-jet events	
Transverse momentum of the ${\text {Z}}$ boson ($j_1$)	$p_{\mathrm {T1}} > 80\,\text {GeV}$, $|y_1 | < 2$
Transverse momentum and rapidity of $$j_2$$	$$p_{\mathrm {T2}} > 80 \,\text {GeV} $$ , $$|y_{2} | < 1 $$
Transverse momentum and rapidity of $$j_3$$	$$p_{\mathrm {T3}} > 20 \,\text {GeV} $$, $$|y_{3} | < 2.4 $$
Azimuthal angle difference between ${\text {Z}}$ and $j_2$	$2< |\varDelta \phi _{12} | < \pi$
Dimuon mass	$70< m_{\mu ^+\mu ^-} < 110 \,\text {GeV}$
Angular distance between $j_3$ and $j_2$	$0.5< \varDelta R_{23} < 1.5$
Number of selected events	15 466

Generator jets are reconstructed from stable particles by clustering the four-vectors with an anti-$k_{\mathrm {T}}$ clustering algorithm excluding neutrinos. The kinematical rerquirements for muons and jets are the same as applied for reconstructed objects. For ${\text {Z}}$ + two-jet events, the distance between muons from ${\text {Z}}$ boson and jets must have $\varDelta R > 0.5$. The $p_{\mathrm {T}} ^\text {miss}$ selection is not applied at the generator level for QCD multijet events.

## Theoretical models

Reconstructed data are compared to predictions from MC event generators, where the generated events are passed through a full detector simulation based on Geant4 [22] and the simulated events are reconstructed using standard CMS software. Reconstruction-level predictions are obtained for three-jet events at $\sqrt{s}= 8\,{\text {TeV}}$ with the MadGraph [23] software package matched to pythia  6 [24] with the CTEQ6L1 [25] parton distribution function (PDF) set and the Z2Star tune [26], as well as with standalone pythia  8.1 [27] with the CTEQ6L1 PDF set and the 4C [28] tune. At 13$\,{\text {TeV}}$, MadGraph interfaced to pythia  8.2 [29] and standalone pythia  8.2 are used with the NNPDF2.3LO [30] PDF set and the CUETP8M1 [31] tune. The sherpa [32] event generator interfaced to csshower++ [33] with the CT10 [34] PDF set and the AMISIC++ [35] tune and MadGraph interfaced to pythia  6 with the CTEQ6L1 PDF set and the Z2Star tune provide ${\text {Z}}$ + two-jet events at 8$\,{\text {TeV}}$. Table 2 summarizes the event generator versions, PDF sets and tunes.

**Table 2:** Event generator versions, PDF sets, and tunes used to produce MC samples at reconstruction level

Event generator	PDF set	Tune
Three-jet events at $\sqrt{s} = 8\,{\text {TeV}}$		
MadGraph 5.1.3.30 + pythia 6.425	CTEQ6L1	Z2Star
pythia 8.153	CTEQ6L1	4C
Three-jet events at $\sqrt{s} = 13\,{\text {TeV}}$		
MadGraph 5.2.3.3 + pythia 8.219	NNPDF2.3LO	CUETP8M1
pythia 8.219	NNPDF2.3LO	CUETP8M1
${\text {Z}}$ + two-jet events		
sherpa 1.4.0 + csshower++	CT10	AMISIC++
MadGraph 5.1.3.30 + pythia 6.425	CTEQ6L1	Z2Star

Results corrected to stable-particle level are compared to predictions obtained with the models presented below. An overview of these models is given in Table 3.

The pythia  8 [29] event generator provides hard-scattering events using a ME calculated at LO supplemented with PS. These event samples are labeled as “pythia LO 2j+PS” for the three-jet and as “pythia LO Z+1j+PS” for ${\text {Z}}$ + two-jet events. The PDF set NNPDF2.3LO and the CUETP8M1 parameter set for the simulation of the underlying event (UE) are used with free parameters adjusted to measurements in ${\text {p}}{\text {p}}$ collisions at the LHC and proton-antiproton collisions at the Fermilab Tevatron. The Lund string model [36] is applied for the hadronization process.

The MadGraph 5_amc@nlo event generator, labeled as “MadGraph ” in the following, is used to simulate hard processes with up to 4 final-state partons at LO accuracy. It is interfaced to pythia  8 with the CUETP8M1 tune and the NNPDF2.3LO PDF set for the simulation of PS, hadronization, and MPI, for three-jet, and to pythia  6 with the Z2Star tune and the CTEQ6L1 PDF set for ${\text {Z}}$ + two-jet events. The three-jet sample is labeled as “MadGraph LO 4j+PS” and the ${\text {Z}}$ + two-jet sample is labeled as “MadGraph LO Z+4j+PS”. The $k_{\mathrm {T}}$-MLM procedure [37] is used to match jets from the ME and PS with a matching scale of 10$\,\text {GeV}$.

Predictions are also included using the powheg box library [38–40], with the CT10 NLO [34] PDFs and with the pythia  8 CUETP8M1 tune applied to simulate PS, MPI, and hadronization. The powheg generator is run in the dijet mode [41] providing an NLO $2\rightarrow 2$ calculation, labeled as “powheg NLO 2j+PS”. The matching between the powheg ME calculations and the pythia UE [31] simulation is performed using the shower-veto procedure (UserHook option 2 [29]).

The sherpa software package is used to simulate ${\text {Z}}$ + two-jet events. The hard process is calculated at LO for a ME with up to four final-state partons and the CT10 PDF set is used. This sample is labeled as “sherpa LO Z+4j+PS”. The sherpa generator has its own PS [33], hadronization, and MPI tune [35].

Finally, the MadGraph 5_amc@nlo generator is also used in the mc@nlo mode, providing a ${\text {Z}}$ + one-jet ME at NLO accuracy. This event generator is interfaced to pythia  8, using the CUETP8M1 tune and the NNPDF3.0NLO [42] PDF set, to produce ${\text {Z}}$ + two-jet events. The sample is labeled as “amc@nlo NLO Z+1j+PS”.

The background from ${\text {W}}$, ${\text {Z}}$, top quark, and diboson production for the three-jet analysis is negligible and not further considered. The main background for ${\text {Z}}$ + two-jet events comes from ${\mathrm{t}\overline{{ \mathrm t}}}$, single top, and diboson production. The ${\mathrm{t}\overline{{ \mathrm t}}}$, ZZ, and WZ events are simulated with MadGraph 5.1.3.30 + pythia 6.425 using the same tune and PDF set as for generating ${\text {Z}}$ + two-jet samples. WW events are generated with pythia 6.425 with CTEQ6L1 PDF set and Z2Star tune. Single top events are generated with powheg (CT10 PDF set, Z2Star tune).

**Table 3:** MC event generators and version numbers, parton-level processes, PDF sets, and UE tunes used for the comparison with measurements

Event generator	Parton-level process	PDF set	Tune
Three-jet events			
pythia 8.219	LO 2j+PS	NNPDF2.3LO	CUETP8M1
MadGraph 5.2.3.3 + pythia 8.219	LO 4j+PS	NNPDF2.3LO	CUETP8M1
powheg 2 + pythia 8.219	NLO 2j+PS	CT10 NLO	CUETP8M1
${\text {Z}}$ + two-jet events			
pythia 8.219	LO Z+1j+PS	NNPDF2.3LO	CUETP8M1
MadGraph 5.1.3.30 + pythia 6.425	LO Z+4j+PS	CTEQ6L1	Z2Star
sherpa 1.4.0 + csshower++	LO Z+4j+PS	CT10	AMISIC++
amc@nlo + pythia 8.223	NLO Z+1j+PS	NNPDF30_nlo_nf_5_pdfas	CUETP8M1

## Data correction and study of systematic uncertainties

To facilitate the comparison of data with theory, the data are unfolded from reconstruction to stable-particle level, defined by a mean decay length larger than 1$\,{\text {cm}}$, so that measurement effects are removed and that the true distributions in the observables are determined. The unfolding is performed using the D’Agostini algorithm [43] as implemented in the RooUnfold software package [44] for three-jet events, while the singular value decomposition method [45] is used for ${\text {Z}}$ + two-jet events. The response matrices are obtained from the full detector simulation using MadGraph for three-jet events and sherpa for ${\text {Z}}$ + two-jet events.

We estimate the influence of ${\mathrm{t}\overline{{ \mathrm t}}}$, single top, and diboson backgrounds by adding generated events produced with event generator MadGraph LO Z+4j+PS and comparing the predictions for the observables $p_{\mathrm {T3}}/p_{\mathrm {T2}}$ and $\varDelta R_{23}$ using the same generator without the backgrounds. For ${\mathrm{t}\overline{{ \mathrm t}}}$ production with fully leptonic decay and dibosons the probability of $j_3$ emission increases from 2% (soft radiation) to 10% (hard radiation) depending on the phase space. For semileptonic and hadronic decays and single top production the change is negligible. Since the background effect is comparable to the systematic uncertainties, it is not included in the theoretical estimations and it is not subtracted from the data.

The distributions are normalized to the integral of the spectra for three-jet events and to the number of inclusive ${\text {Z}}$ + one-jet events in the ${\text {Z}}$ + two-jet analysis. The ${\text {Z}}$ + two-jet analysis normalization thus reflects the probability to have more than one jet in the event.

Systematic uncertainties associated to the jet energy scale (JES) calibration, the jet energy resolution (JER), PU modeling, model dependence, as well as the unfolding method, are estimated. Muon-related uncertainties (single muon trigger efficiency, muon isolation, muon scale and resolution) for the ${\text {Z}}$ + two-jet channel are negligible with respect to other systematic sources. The treatment of the uncertainty depends on the uncertainty source and is estimated separately for each bin (see below). The overall uncertainty for each bin is estimated summing in quadrature uncertainties from the various sources.

The systematic uncertainty from the JES is 0.15 (0.24)% at $\sqrt{s} = 8\,(13)\,{\text {TeV}}$ for the three-jet case and 5–10% for the ${\text {Z}}$ + two-jet events. The JER observed in data differs from that obtained from simulation and simulated jets are therefore smeared to obtain the same resolution as in the data [46]. The systematic uncertainty from JER is estimated by varying the simulated JER uncertainty up and down by one standard deviation, which results in a systematic uncertainty of 0.16 (0.12)% at $\sqrt{s} = 8\,(13)\,{\text {TeV}}$ for three-jet and 2–3% for ${\text {Z}}$ + two-jet events. When the distributions of ${\text {Z}}$ + two-jet events are normalized to the integrals of the histograms, instead of the number of ${\text {Z}}$ + one-jet events, the systematic uncertainties due to the JES and JER decrease to 0.3–0.5%, except for the $p_{\mathrm {T3}}/p_{\mathrm {T2}}$ shape, which is still sensitive to the JES with changes of up to 3%.

The distribution in the number of primary vertices is sensitive to the PU difference between data and simulation. To estimate the uncertainty due to the PU modeling, the number of PU events in simulation is changed by shifting the total inelastic cross section by ±5% [47]. The resulting PU uncertainties are 0.10 (0.17)% at $\sqrt{s} = 8\,(13)\,{\text {TeV}}$ for the three-jet and 1% for the ${\text {Z}}$ + two-jet events.

The dependence on the event generator used for the unfolding is estimated with MC event samples from MadGraph and pythia for three-jet, and sherpa and MadGraph for the ${\text {Z}}$ + two-jet events. The means of both sets of unfolded data are used as the nominal values. This uncertainty is $\approx 1.1$ (0.25)% at $\sqrt{s} = 8\,(13)\,{\text {TeV}}$ for the three-jet and 1% for the ${\text {Z}}$ + two-jet events, which is half of the difference between the results obtained with the respective event generators. The difference in the results is due to statistical fluctuations from the limited number of events in the MC simulation.

Table 4 summarizes the systematic uncertainties in the measurements.

**Table 4:** Systematic uncertainties in the measurements in %

Source	three-jet 8/13$\,{\text {TeV}}$	${\text {Z}}$ + two-jet 8$\,{\text {TeV}}$
Jet energy scale	0.15/0.24	5–10
Jet energy resolution	0.16/0.12	2–3
Pileup	0.1/0.17	1
Unfolding and model dependence	1.1/0.25	1

The systematic uncertainties from various sources are similar for the three-jet samples at $\sqrt{s} = 8$ and 13 $\,{\text {TeV}}$, except for unfolding and model dependence at $\sqrt{s} = 8\,{\text {TeV}}$. The systematic uncertainties between the three-jet and ${\text {Z}}$ + two-jet analysis cannot be compared directly because each analysis uses a different normalization and also differs in statistical significance. The JES uncertainty is especially sensitive to the jet $p_{\mathrm {T}}$ range, and the ${\text {Z}}$ + two-jet phase space has a lower $p_{\mathrm {T}}$ threshold than the one used in the three-jet events.

The figures of Sect. 6 show the total systematic uncertainty as a band in the panels displaying the ratio of predictions over data.

## Results

We compare the distributions in the ratio $p_{\mathrm {T3}}/p_{\mathrm {T2}}$ in data to predictions for events with small-angle ($\varDelta R_{23} < 1.0$) and large-angle radiation ($\varDelta R_{23} > 1.0$). We also compare the $\varDelta R_{23}$ distributions in data to predictions with soft ($p_{\mathrm {T3}}/p_{\mathrm {T2}} < 0.3$) and hard radiation ($p_{\mathrm {T3}}/p_{\mathrm {T2}} > 0.6$). The events with $0.3< p_{\mathrm {T3}}/p_{\mathrm {T2}} < 0.6$ are not used in the comparisons for the $\varDelta R_{23}$ observable because we focus on the limits in soft and hard radiation. This classification is summarized in Fig. 1, within the phase space defined in Table 1. The data measurements are provided at the Durham High Energy Physics Database (HEPData) [48].

**Fig. 2:**
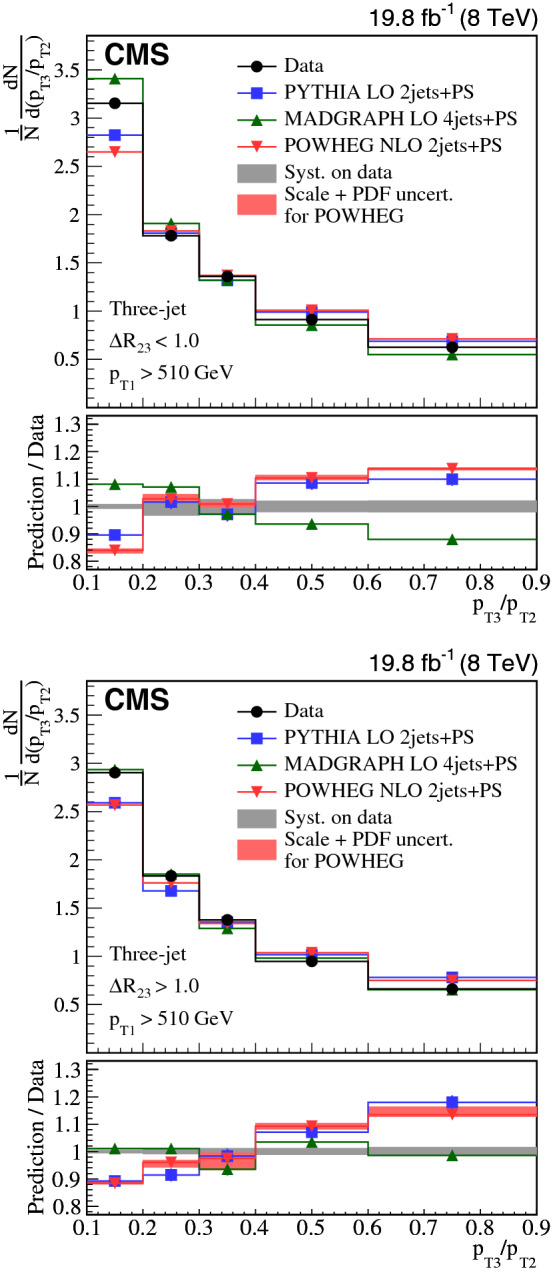
Three-jet events at $\sqrt{s} = 8\,{\text {TeV}}$ compared to theory: (upper) $p_{\mathrm {T3}}/p_{\mathrm {T2}}$ for small-angle radiation ($\varDelta R_{23} < 1.0$), (lower) $p_{\mathrm {T3}}/p_{\mathrm {T2}}$ for large-angle radiation ($\varDelta R_{23} > 1.0$)

**Fig. 3:**
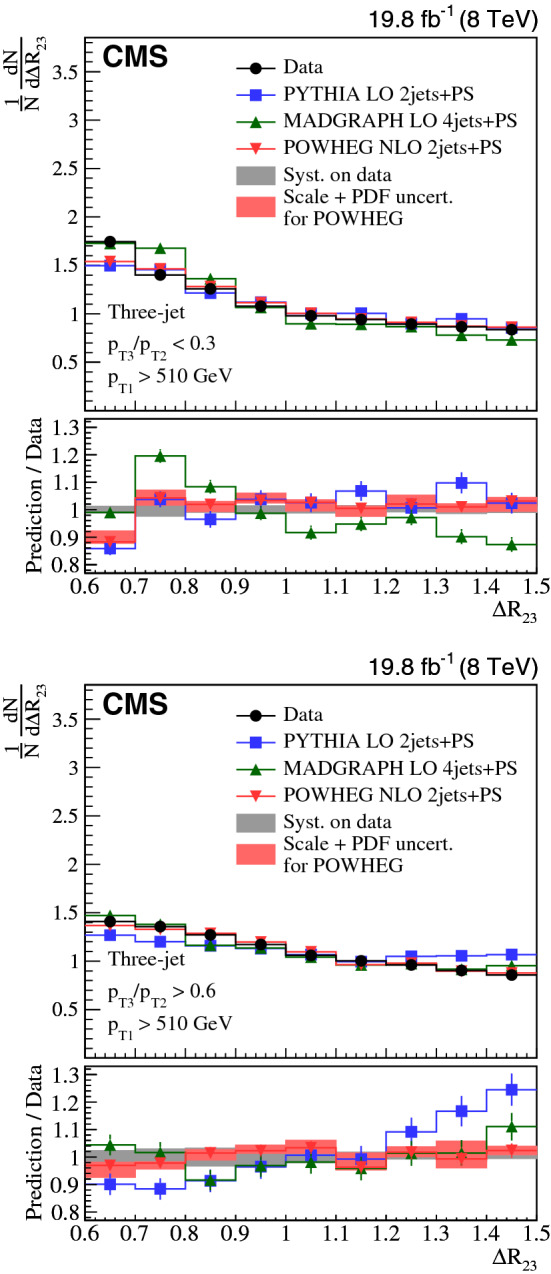
Three-jet events at $\sqrt{s} = 8\,{\text {TeV}}$ and comparison to theoretical predictions: (upper) $\varDelta R_{23}$ for soft radiation ($p_{\mathrm {T3}}/p_{\mathrm {T2}} < 0.3$), (lower) $\varDelta R_{23}$ for hard radiation ($p_{\mathrm {T3}}/p_{\mathrm {T2}} > 0.6$)

**Fig. 4:**
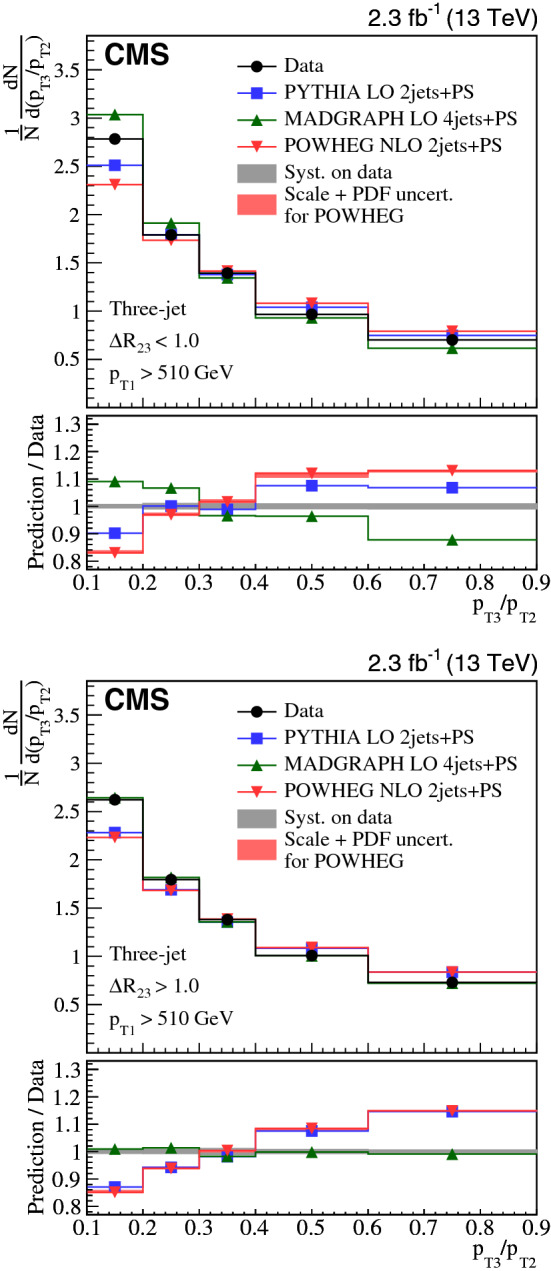
Three-jet events at $\sqrt{s} = 13\,{\text {TeV}}$ compared to theory: (upper) $p_{\mathrm {T3}}/p_{\mathrm {T2}}$ for small-angle radiation ($\varDelta R_{23} < 1.0$), (lower) $p_{\mathrm {T3}}/p_{\mathrm {T2}}$ for large-angle radiation ($\varDelta R_{23} > 1.0$)

**Fig. 5:**
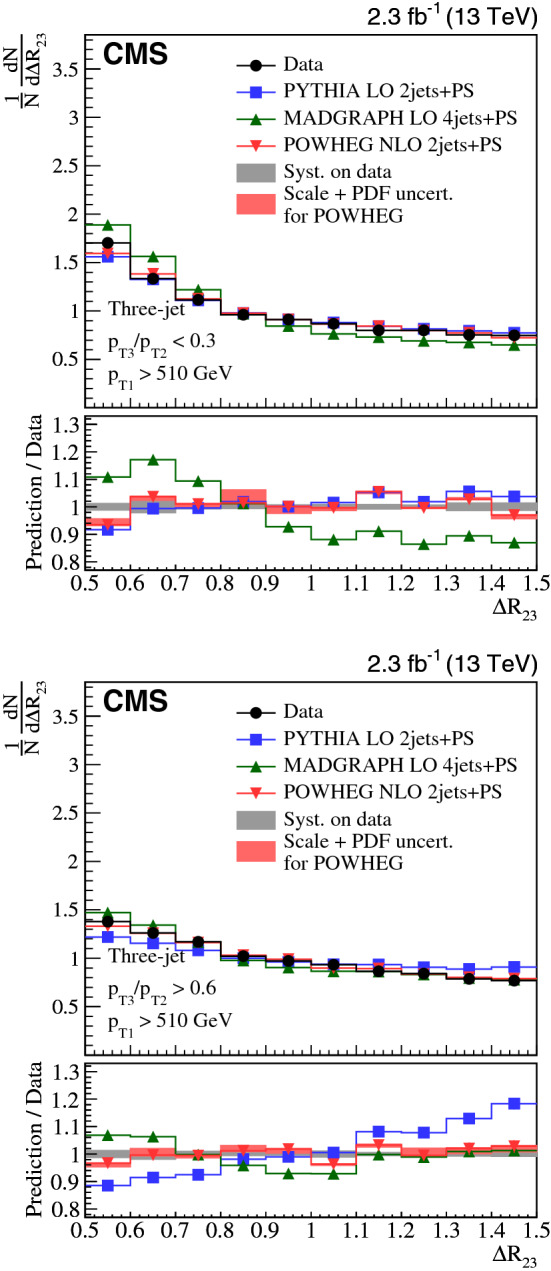
Three-jet events at $\sqrt{s} = 13\,{\text {TeV}}$ and comparison to theoretical predictions: (upper) $\varDelta R_{23}$ for soft radiation ($p_{\mathrm {T3}}/p_{\mathrm {T2}} < 0.3$), (lower) $\varDelta R_{23}$ for hard radiation ($p_{\mathrm {T3}}/p_{\mathrm {T2}} > 0.6$)

**Fig. 6:**
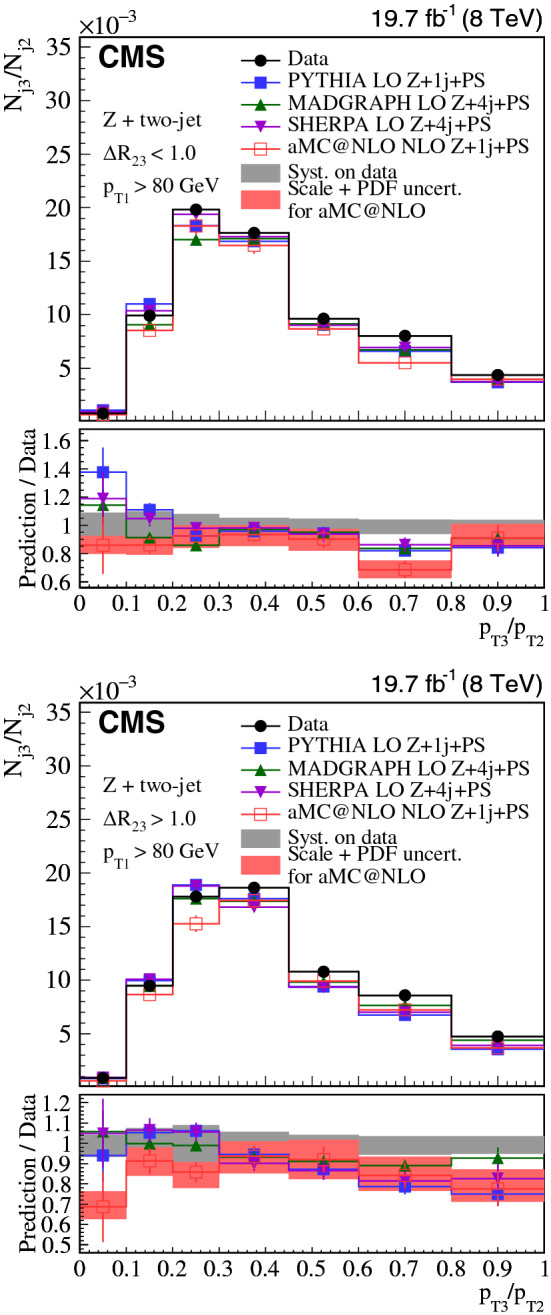
${\text {Z}}$ + two-jet events at $\sqrt{s} = 8\,{\text {TeV}}$ compared to theory: (upper) $p_{\mathrm {T3}}/p_{\mathrm {T2}}$ for small-angle radiation ($\varDelta R_{23} < 1.0$), (lower) $p_{\mathrm {T3}}/p_{\mathrm {T2}}$ for large-angle radiation ($\varDelta R_{23} > 1.0$)

**Fig. 7:**
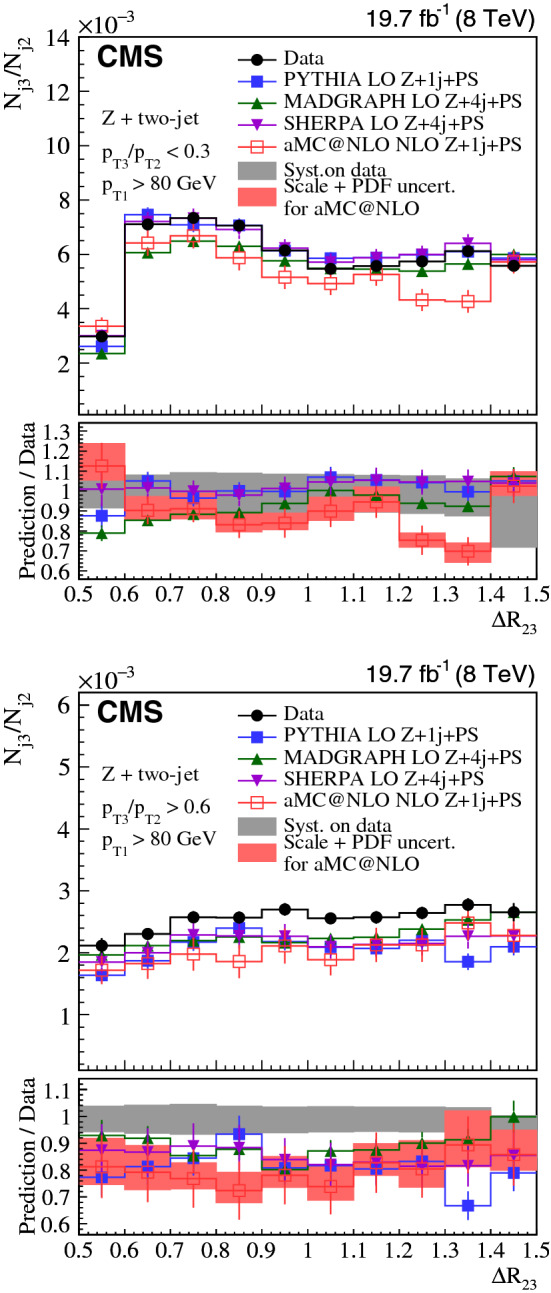
${\text {Z}}$ + two-jet events at $\sqrt{s} = 8\,{\text {TeV}}$ compared to theory: (upper) $\varDelta R_{23}$ for soft radiation ($p_{\mathrm {T3}}/p_{\mathrm {T2}} < 0.3$), (lower) $\varDelta R_{23}$ for hard radiation ($p_{\mathrm {T3}}/p_{\mathrm {T2}} > 0.6$)

The uncertainties in the PDF and in the renormalization and factorization scales are investigated for the powheg and amc@nlo models. Other theoretical predictions are expected to have comparable uncertainties. The PDF uncertainties are calculated as recommended in PDF4LHC [49] following the description of the PDF sets: for CT10 using the Hessian approach; and for NNPDF using MC replicas. The renormalization and factorization scales are varied by a factor 2 up and down, excluding the (2,1/2) and (1/2,2) cases. Finally, the theoretical uncertainties are obtained as the quadratic sum of the PDF variance and the envelope of the scale variations, and displayed as a band around the theoretical predictions in the Figs. 2, 3, 4, 5, 6 and 7.

### Three-jet selection

We show the $\sqrt{s}=8\,{\text {TeV}}$ measurements of $p_{\mathrm {T3}}/p_{\mathrm {T2}}$ in Fig. 2 and of $\varDelta R_{23}$ in Fig. 3, and compare them to theoretical expectations. In Figs. 4 and 5 the distributions are given for $\sqrt{s} = 13\,{\text {TeV}}$. Figure 2 (upper) shows the $p_{\mathrm {T3}}/p_{\mathrm {T2}}$ distribution for the small $\varDelta R_{23}$ region. All predictions show significant deviations from the measurements. Interestingly, the LO 4j+PS prediction shows different behavior compared with LO 2j+PS and NLO 2j+PS. We see that the number of partons in the ME calculation and the merging method with the PS in the present simulations lead to different predictions. In Fig. 2 (lower) the $p_{\mathrm {T3}}/p_{\mathrm {T2}}$ distribution is shown for large $\varDelta R_{23}$. This region of phase space is well described by the LO 4j+PS calculations, while the LO 2j+PS and NLO 2j+PS predictions show large deviations from the measurements.

In Fig. 3, the $\varDelta R_{23}$ distribution is shown for two regions of $p_{\mathrm {T3}}/p_{\mathrm {T2}}$. Figure 3 (upper) shows $p_{\mathrm {T3}}/p_{\mathrm {T2}} < 0.3$. The predictions from LO 2j+PS and NLO 2j+PS describe the measurement well, while the prediction from LO 4j+PS shows a larger deviation from the data. In Fig. 3 (lower) the $\varDelta R_{23}$ distribution is shown for $p_{\mathrm {T3}}/p_{\mathrm {T2}} > 0.6$. In contrast to Fig. 3 (upper), the predictions for distributions from LO 2j+PS differ from the measurement, whereas the predictions from NLO 2j+PS and LO 4j+PS agree well with it. This indicates that in this region the contribution from higher-multiplicity ME calculations supplemented with PS should be included. The same comparisons are performed for the $\sqrt{s} = 13\,{\text {TeV}}$ measurements as shown in Figs. 4 and 5. A similar behavior is observed for $\sqrt{s} = 8\,{\text {TeV}}$. In conclusion, none of the simulations simultaneously describes to simultaneously describe both the $p_{\mathrm {T3}}/p_{\mathrm {T2}}$ and the $\varDelta R_{23}$ distributions in three-jet events.

### ${\text {Z}}$ + two-jet selection

The measurement of $p_{\mathrm {T3}}/p_{\mathrm {T2}}$ for ${\text {Z}}$ + two-jet events is presented in Fig. 6 for data at $\sqrt{s} = 8\,{\text {TeV}}$. All distributions are normalized to the selected number of ${\text {Z}}$ + one-jet events. All predictions from pythia, sherpa, MadGraph, and amc@nlo agree with data within the uncertainties of the measurement except for the phase space region with hard radiation.

Figure 7 shows the measurement as a function of $\varDelta R_{23}$. The amc@nlo prediction deviates from the data at high $\varDelta R_{23}$ and small $p_{\mathrm {T3}}/p_{\mathrm {T2}}$, while pythia, sherpa, MadGraph, and amc@nlo describe the shape of the distribution in the high-$p_{\mathrm {T3}}/p_{\mathrm {T2}}$ range, but underestimate the data due to a smaller contribution from production of $j_3$. This feature is based on the normalization of ${\text {Z}}$ + two-jet distributions by the number of inclusive ${\text {Z}}$ + one-jet events selected.

**Fig. 8:**
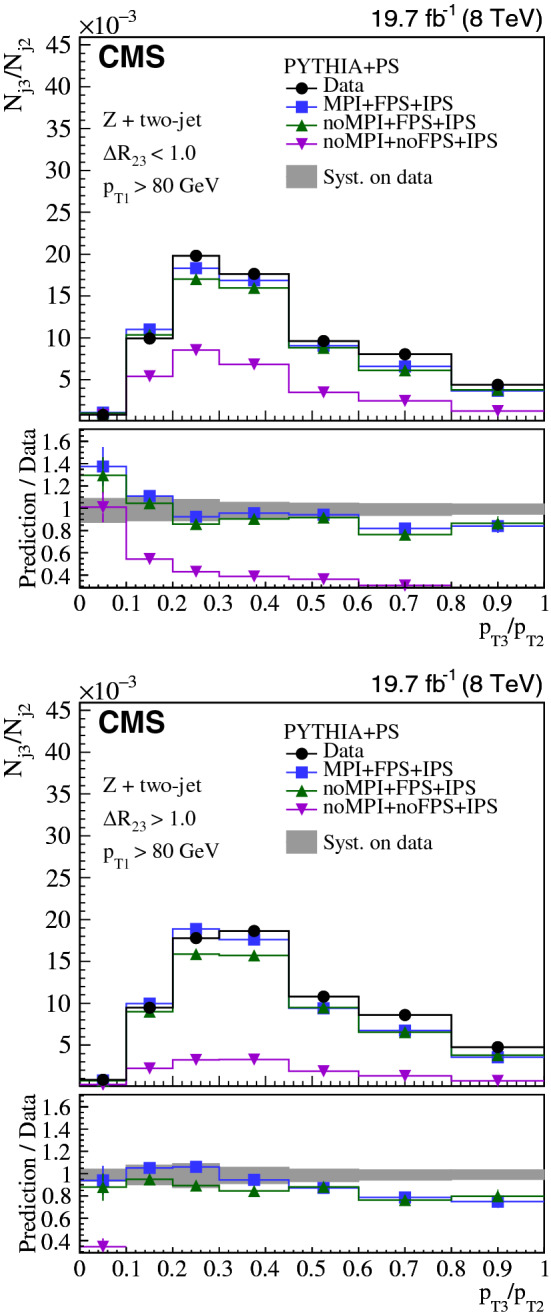
${\text {Z}}$ + two-jet events at $\sqrt{s} = 8\,{\text {TeV}}$ compared to theoretical predictions from pythia  8 without initial-state parton showers (IPS), final-state parton showers (FPS), and MPI: (upper) $p_{\mathrm {T3}}/p_{\mathrm {T2}}$ for small-angle radiation ($\varDelta R_{23} < 1.0$), (lower) $p_{\mathrm {T3}}/p_{\mathrm {T2}}$ for large-angle radiation ($\varDelta R_{23} > 1.0$)

**Fig. 9:**
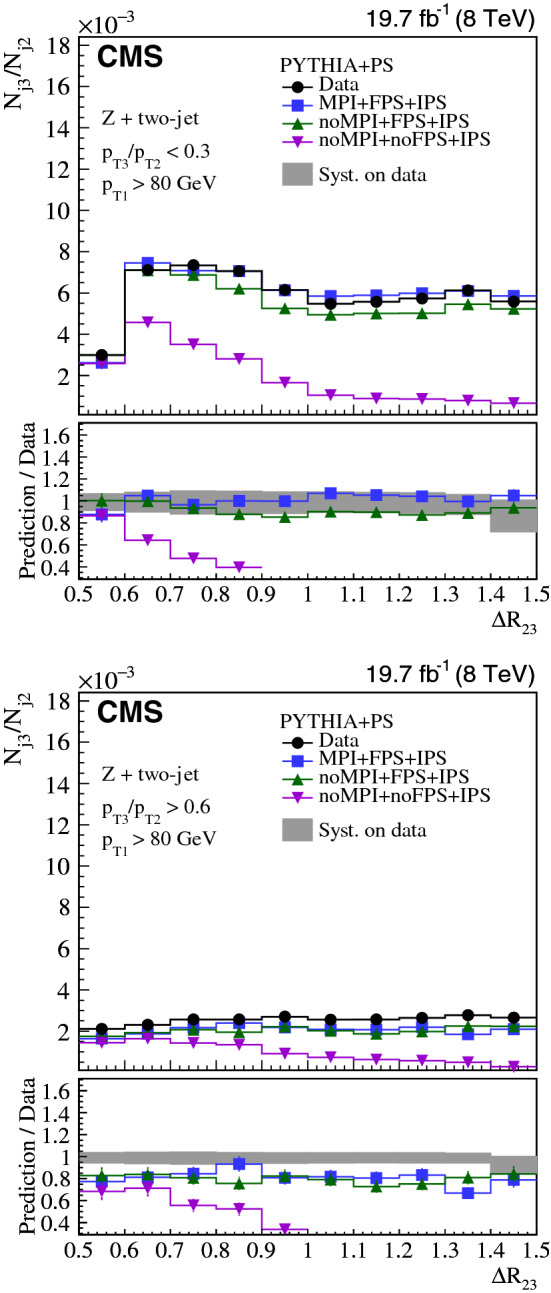
${\text {Z}}$ + two-jet events at $\sqrt{s} = 8\,{\text {TeV}}$ and comparison to theoretical predictions from pythia  8 without initial-state parton showers (IPS), final-state parton showers (FPS), and MPI: (upper) $\varDelta R_{23}$ for soft radiation ($p_{\mathrm {T3}}/p_{\mathrm {T2}} < 0.3$), (lower) $\varDelta R_{23}$ for hard radiation ($p_{\mathrm {T3}}/p_{\mathrm {T2}} > 0.6$).

Figures 8 and 9 compare the event distributions with predictions from pythia  8 with the final-state PS and MPI switched off. The initial-state PS was kept, because one of the jets must originate from PS when ${\text {Z}}$ + two-jet events are selected. Multiple parton interactions play a very minor role, while the final-state PS in pythia  8 is very important. When the final-state PS is switched off, events where both jets come from the initial-state PS are kept with a tendency to be close to each other in $\varDelta R_{23}$.

In general, the measurements with ${\text {Z}}$ + two-jet events are well described by all theoretical predictions, except for the underestimation of the $j_3$ emission. The contribution of background from ${\mathrm{t}\overline{{ \mathrm t}}}$ production and dibosons can partially compensate the lack of the $j_3$ emission. The contribution of the background (${\mathrm{t}\overline{{ \mathrm t}}}$ production with fully leptonic decay and dibosons) increases the probability of $j_3$ emission from 2% (soft radiation) to 10% (hard radiation) depending on the phase space region. The effect of the other processes (${\mathrm{t}\overline{{ \mathrm t}}}$ production with semileptonic and hadronic decays, single top production) is negligible. In comparison with the three-jet measurements, we observe significant differences; only in the region of large $\varDelta R_{23}$ and large $p_{\mathrm {T3}}/p_{\mathrm {T2}}$ (hard and large-angle radiation) do the theoretical predictions agree with the measurement. The accessible range in $p_{\mathrm {T}}$ is rather small in ${\text {Z}}$ + two-jet events because of the limit in the $p_{\mathrm {T}}$ of the ${\text {Z}}$ bosons ($p_{\mathrm {T1}} > 80\,\text {GeV}$), while the three-jet selection, on the contrary, can have a rather large range ($p_{\mathrm {T1}} > 510\,\text {GeV}$). This may explain why the region of small $p_{\mathrm {T3}}/p_{\mathrm {T2}}$ is better described by predictions that include PS in the latter case. In addition, the large-angle radiation is best described by fixed-order ME calculations.

In conclusion, the ${\text {Z}}$ + two-jet measurement has a different distribution in $p_{\mathrm {T3}}/p_{\mathrm {T2}}$, which originates from the different kinematic selection criteria relative to three-jet events, thus reducing the sensitivity in the soft and collinear region. Within the available phase space, the measurements are in reasonable agreement with both PS and ME calculations, apart from the emission of $j_3$ in the high-$p_{\mathrm {T3}}/p_{\mathrm {T2}}$ region.

## Summary

Two kinematic variables are introduced to quantify the radiation pattern in multijet events: (i) the transverse momentum ratio ($p_{\mathrm {T3}}/p_{\mathrm {T2}}$) of two jets, and (ii) their angular separation ($\varDelta R_{23}$). The variable $p_{\mathrm {T3}}/p_{\mathrm {T2}}$ is used to distinguish between soft and hard radiation, while $\varDelta R_{23}$ classifies events into small- and large-angle radiation types. Events with three or more energetic jets as well as inclusive ${\text {Z}}$ + two-jet events are selected for study using data collected at $\sqrt{s} = 8\,{\text {TeV}}$ corresponding to an integrated luminosity of 19.8$\,\text {fb}^{-1}$. Three-jet events at $\sqrt{s} = 13\,{\text {TeV}}$ corresponding to an integrated luminosity of 2.3$\,\text {fb}^{-1}$ are also analyzed. No significant dependence on the center-of-mass energy is observed in the differential distributions of $p_{\mathrm {T3}}/p_{\mathrm {T2}}$ and $\varDelta R_{23}$.

Overall, large-angle radiation (large $\varDelta R_{23}$) and hard radiation (large $p_{\mathrm {T3}}/p_{\mathrm {T2}}$) are well described by the matrix element (ME) calculations (using LO 4j+PS formulations), while the parton shower (PS) approach (LO 2j+PS and NLO 2j+PS) fail to describe the regions of large-angle and hard radiation. The collinear region (small $\varDelta R_{23}$) is not well described; LO 2j+PS, NLO 2j+PS, and LO 4j+PS distributions show deviations from the measurements. In the soft region (small $p_{\mathrm {T3}}/p_{\mathrm {T2}}$), the PS approach describes the measurement also in the large-angle region (full range in $\varDelta R_{23}$), while for large $p_{\mathrm {T3}}/p_{\mathrm {T2}}$ higher-order ME contributions are needed to describe the three-jet measurements. The distributions in ${\text {Z}}$ + two-jet events are reasonably described by all tested generators. Nevertheless, we find an underestimation of third-jet emission at large $p_{\mathrm {T3}}/p_{\mathrm {T2}}$ both in the collinear and large-angle regions, for all of the tested models. Contribution from ${\mathrm{t}\overline{{ \mathrm t}}}$ and dibosons production may partially cover the difference. These results illustrate how well the collinear/soft, and large-angle/hard regions are described by different approaches. The different kinematic regions and initial-state flavor composition may be the reason why the three-jet measurements are less consistent with the theoretical predictions relative to the ${\text {Z}}$ + two-jet final states. These results clearly indicate that the methods of merging ME with PS calculations are not yet optimal for describing the full region of phase space. The measurements presented here serve as benchmarks for future improved predictions coming from ME calculations combined with parton showers.

## Data Availability

This manuscript has no associated data or the data will not be deposited. [Authors’ comment: Release and preservation of data used by the CMS Collaboration as the basis for publications is guided by the CMS policy as written in its document “CMS data preservation, re-use and open access policy” ( https://cms-docdb.cern.ch/cgi-bin/PublicDocDB/RetrieveFile?docid=6032&filename=CMSDataPolicyV1.2.pdf&version=2 ).]
